# The GAAS Metagenomic Tool and Its Estimations of Viral and Microbial Average Genome Size in Four Major Biomes

**DOI:** 10.1371/journal.pcbi.1000593

**Published:** 2009-12-11

**Authors:** Florent E. Angly, Dana Willner, Alejandra Prieto-Davó, Robert A. Edwards, Robert Schmieder, Rebecca Vega-Thurber, Dionysios A. Antonopoulos, Katie Barott, Matthew T. Cottrell, Christelle Desnues, Elizabeth A. Dinsdale, Mike Furlan, Matthew Haynes, Matthew R. Henn, Yongfei Hu, David L. Kirchman, Tracey McDole, John D. McPherson, Folker Meyer, R. Michael Miller, Egbert Mundt, Robert K. Naviaux, Beltran Rodriguez-Mueller, Rick Stevens, Linda Wegley, Lixin Zhang, Baoli Zhu, Forest Rohwer

**Affiliations:** 1 Biology Department, San Diego State University, San Diego, California, United States of America; 2 Computational Science Research Center, San Diego State University, San Diego, California, United States of America; 3 Computer Science Department, San Diego State University, San Diego, California, United States of America; 4 Mathematics and Computer Science Division, Argonne National Lab, Argonne, Illinois, United States of America; 5 Biosciences Division, Argonne National Laboratory, Argonne, Illinois, United States of America; 6 Biology Department, Florida International University, Miami, Florida, United States of America; 7 School of Marine Science and Policy, University of Delaware, Lewes, Delaware, United States of America; 8 URMITE, Centre National de la Recherche Scientifique UMR IRD 6236, Université de la Méditerranée, Marseille, France; 9 The Broad Institute of Massachusetts Institute of Technology and Harvard, Cambridge, Massachusetts, United States of America; 10 CAS Key Laboratory of Pathogenic Microbiology and Immunology, Institute of Microbiology, Chinese Academy of Sciences, Beijing, China; 11 Ontario Institute for Cancer Research, MaRS Centre, Toronto, Ontario, Canada; 12 Poultry Diagnostic and Research Center, College of Veterinary Medicine, The University of Georgia, Athens, Georgia, United States of America; 13 School of Medicine, University of California San Diego, San Diego, United States of America; Washington University School of Medicine, United States of America

## Abstract

Metagenomic studies characterize both the composition and diversity of uncultured viral and microbial communities. BLAST-based comparisons have typically been used for such analyses; however, sampling biases, high percentages of unknown sequences, and the use of arbitrary thresholds to find significant similarities can decrease the accuracy and validity of estimates. Here, we present Genome relative Abundance and Average Size (GAAS), a complete software package that provides improved estimates of community composition and average genome length for metagenomes in both textual and graphical formats. GAAS implements a novel methodology to control for sampling bias via length normalization, to adjust for multiple BLAST similarities by similarity weighting, and to select significant similarities using relative alignment lengths. In benchmark tests, the GAAS method was robust to both high percentages of unknown sequences and to variations in metagenomic sequence read lengths. Re-analysis of the Sargasso Sea virome using GAAS indicated that standard methodologies for metagenomic analysis may dramatically underestimate the abundance and importance of organisms with small genomes in environmental systems. Using GAAS, we conducted a meta-analysis of microbial and viral average genome lengths in over 150 metagenomes from four biomes to determine whether genome lengths vary consistently between and within biomes, and between microbial and viral communities from the same environment. Significant differences between biomes and within aquatic sub-biomes (oceans, hypersaline systems, freshwater, and microbialites) suggested that average genome length is a fundamental property of environments driven by factors at the sub-biome level. The behavior of paired viral and microbial metagenomes from the same environment indicated that microbial and viral average genome sizes are independent of each other, but indicative of community responses to stressors and environmental conditions.

## Author Summary

Metagenomics uses DNA or RNA sequences isolated directly from the environment to determine what viruses or microorganisms exist in natural communities and what metabolic activities they encode. Typically, metagenomic sequences are compared to annotated sequences in public databases using the BLAST search tool. Our methods, implemented in the Genome relative Abundance and Average Size (GAAS) software, improve the way BLAST searches are processed to estimate the taxonomic composition of communities and their average genome length. GAAS provides a more accurate picture of community composition by correcting for a systematic sampling bias towards larger genomes, and is useful in situations where organisms with small genomes are abundant, such as disease outbreaks caused by small RNA viruses. Microbial average genome length relates to environmental complexity and the distribution of genome lengths describes community diversity. A study of the average genome length of viruses and microorganisms in four different biomes using GAAS on 169 metagenomes showed significantly different average genome sizes between biomes, and large variability within biomes as well. This also revealed that microbial and viral average genome sizes in the same environment are independent of each other, which reflects the different ways that microorganisms and viruses respond to stress and environmental conditions.

## Introduction

Metagenomic approaches to the study of microbial and viral communities have revealed previously undiscovered diversity on a tremendous scale [1],[2]. Metagenomic sequences are typically compared to sequences from known genomes using BLAST to estimate the taxonomic and functional composition of the original environmental community [3]. Many software tools designed to estimate community composition (e.g. MEGAN) annotate sequences using only the best similarity [4]. However, the best similarity is often not from the most closely related organism [5]. In addition, most metagenomes contain a large percentage of sequences from novel organisms which cannot be identified by BLAST similarities, further complicating analysis [1],[6],[7].

Mathematical methods based on contig assembly have been developed to estimate viral diversity and community structure from metagenomic sequences regardless of whether they are similar to known sequences [8]. These similarity-independent methods require the input of the average genome length of viruses from a given sample [8]. Having an accurate value of this average is important because it takes a potentially large range spanning 3 orders of magnitude, and has a large influence on the diversity estimates. Average genome length for an environmental community can be determined using Pulsed Field Gel Electrophoresis (PFGE) [9],[10]. PFGE gives a spectrum of genome lengths in a microbial or viral consortium, indicated by electrophoretic bands on an agarose gel, which can be used to calculate an average genome length. Due to the large variability of dsDNA virus genome length, PFGE can discriminate and identify dominant viral populations [11]. However, PFGE is limited because the bands are not independent and a single band can contain different DNA sequences [12],[13].

Average genome length in environmental samples has also been used as a metric to describe community diversity and complexity [9], [14]–[17]. In PFGE, both a larger size range and a greater number of bands indicate a wider variety of genomes and hence, a more diverse community [9],[14],[16],[17]. The average genome length of a microbial community has been shown to serve as a proxy for the complexity of an ecosystem [15]. Longer average genome lengths indicate higher complexity [15], since larger bacterial genomes can encode more genes and access more resources [18].

Here we introduce Genome relative Abundance and Average Size (GAAS), the first bioinformatic software package that simultaneously estimates both genome relative abundance and average genome length from metagenomic sequences. GAAS is implemented in Perl and is freely available at http://sourceforge.net/projects/gaas/. Unlike methods that rely on microbial marker genes to estimate genome length, the GAAS method can be applied to viruses, which lack a universally common genetic element [19]. GAAS determines community composition and average genome length using a novel BLAST-based approach that maintains all similarities with significant relative alignment lengths, assigns them statistical weights, and normalizes by target genome length to calculate accurate relative abundances. Using GAAS, the community composition and average genome length for over 150 viral and microbial metagenomes was derived from four different biomes, including the Sargasso Sea virome previously described in Angly et al. [1]. The average genome lengths were used in a meta-analysis to determine how genome length varies at three levels: between biomes (e.g. terrestrial versus aquatic), between related sub-biomes (e.g. ocean versus freshwater), and between microbial and viral communities sampled from the same environment.

## Results/Discussion

### Accuracy of GAAS estimates

**Figure 1:**
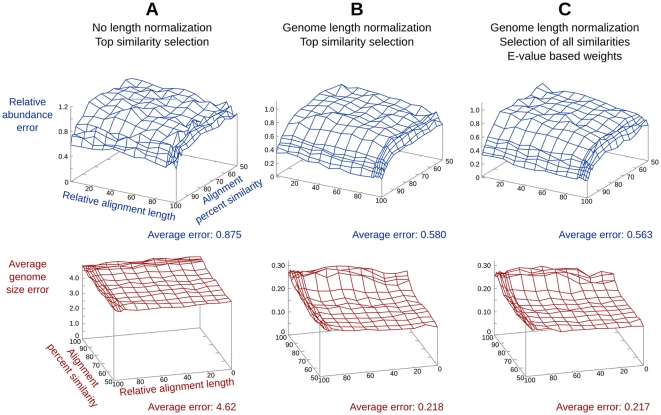
Effects of length normalization and similarity weighting on the accuracy of GAAS estimates. Different methods were used: (A) the standard method (no length normalization, selection of the top similarity only), (B) a combination of genome length normalization and top similarity selection only, and (C) the GAAS method (genome length normalization, selection of all significant similarities, and E-value based weights). Decreases in average error indicate increased accuracy. In the simulated viral metagenomes, 100 bp sequences were used and 80% of the species were considered unknown.

GAAS provided more accurate estimates of average genome length and community composition than standard BLAST searches (i.e. no length normalization, no relative alignment length filtering, top BLAST similarity only) (Figure 1). The accuracy of GAAS estimates was benchmarked using artificial viral metagenomes. To simulate environmental metagenomes, 80% of species were treated as unknowns and viral communities were created with either power law or uniform rank-abundance structures. The error for power law metagenomes was consistently higher than for the uniform case (data not shown). Significance of BLAST similarities was determined using relative alignment length and percentage of similarity in addition to an E-value cutoff. The accuracy of GAAS was dramatically increased by normalizing for genome length; average errors decreased significantly for community composition (p<0.001, Mann-Whitney U test), as well as genome length (p<0.001, Mann-Whitney U test) (Figure 1 A, B). Metagenomes consist of sequence fragments derived from the available genomes in an environment [20]. Even if two genomes are present in equal abundances, a larger genome has a higher probability of being sampled because it will produce more fragments of a given size per genome (Figure S1). Length normalization in GAAS corrected for this sampling bias inherent to the construction of random shotgun libraries such as metagenomes. Using all similarities weighted proportionally to their E-values further reduced errors in composition. This reduction was significant in comparison to average error when only the top BLAST similarity was used (p<0.001, Mann-Whitney U test) (Figure 1 C). When no species were treated as unknown, the error on the GAAS estimates decreased dramatically (Figure S2). GAAS performed well in benchmarks using artificial microbial metagenomes obtained from JGI (Figure S3). Figure S4 shows that it is harder to distinguish between closely related strains than unrelated species using local similarities: the error on the relative abundance estimates is higher than for more distantly related microorganisms (Figure S3). However, GAAS improves both estimates of relative abundance and average genome length, from ∼2% relative error for the average genome size when keeping only the top similarity to ∼0.2% using all similarities and weighting them (Figure S4).

### Read length does not matter for GAAS

**Figure 2:**
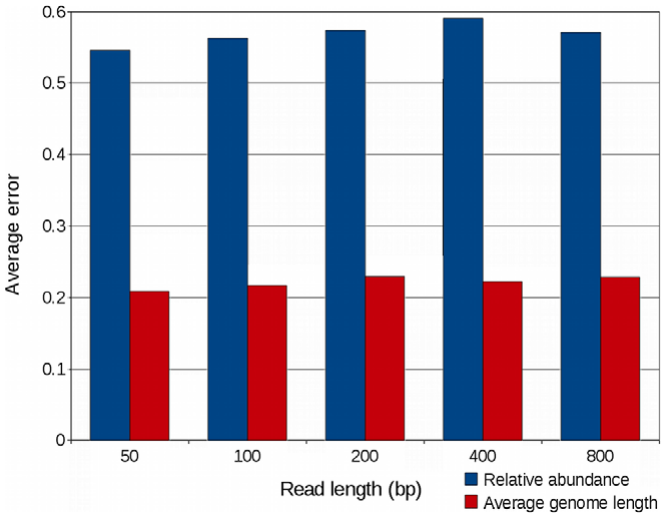
Effects of metagenomic read length on average error of GAAS estimates. Decreases in average error indicate increased accuracy. In the simulated metagenomes, 80% of the species were considered unknown. See Figure S5 and Figure S6 for full details.

Variations in metagenomic read lengths did not affect the accuracy of GAAS relative genome length estimates (Figure 2, Figure S5, Figure S6). GAAS was benchmarked on simulated viral metagenomes containing 50, 100, 200, 400, or 800 base pair sequences. Read length had no effect on the accuracy of average genome length estimates (p = 0.408, Kruskal-Wallis test). Average errors in composition increased significantly (p<0.001, Kruskal-Wallis test) with increasing read length, but there was only a very weak positive correlation between increased errors and longer reads (tau = 0.07, p<0.001). The accuracy of GAAS estimates was thus not very susceptible to changes in read length on average. This contrasts with a report on the inappropriateness of short reads for characterizing environmental communities, mainly on the basis that they miss more distant homologies than longer sequences [21]. In addition, the longest reads tested here (800 bp) achieved both the lowest and highest error on the relative abundance estimates (Figure S5). This indicates that the choice of appropriate filtering parameters is more important for longer sequences than for short sequences. In summary, GAAS can be used to accurately and effectively estimate both composition and average genome length for sequences from a variety of available technologies: very short (∼50 bp) sequences obtained by reversible chain termination sequencing (e.g. Solexa), mid-size sequences produced by Roche 454 pyrosequencing (∼100–400 bp), and long 700+ bp reads sequenced by synthetic chain-terminator chemistry (Sanger).

### Re-analysis of the Sargasso Sea virome

**Figure 3:**
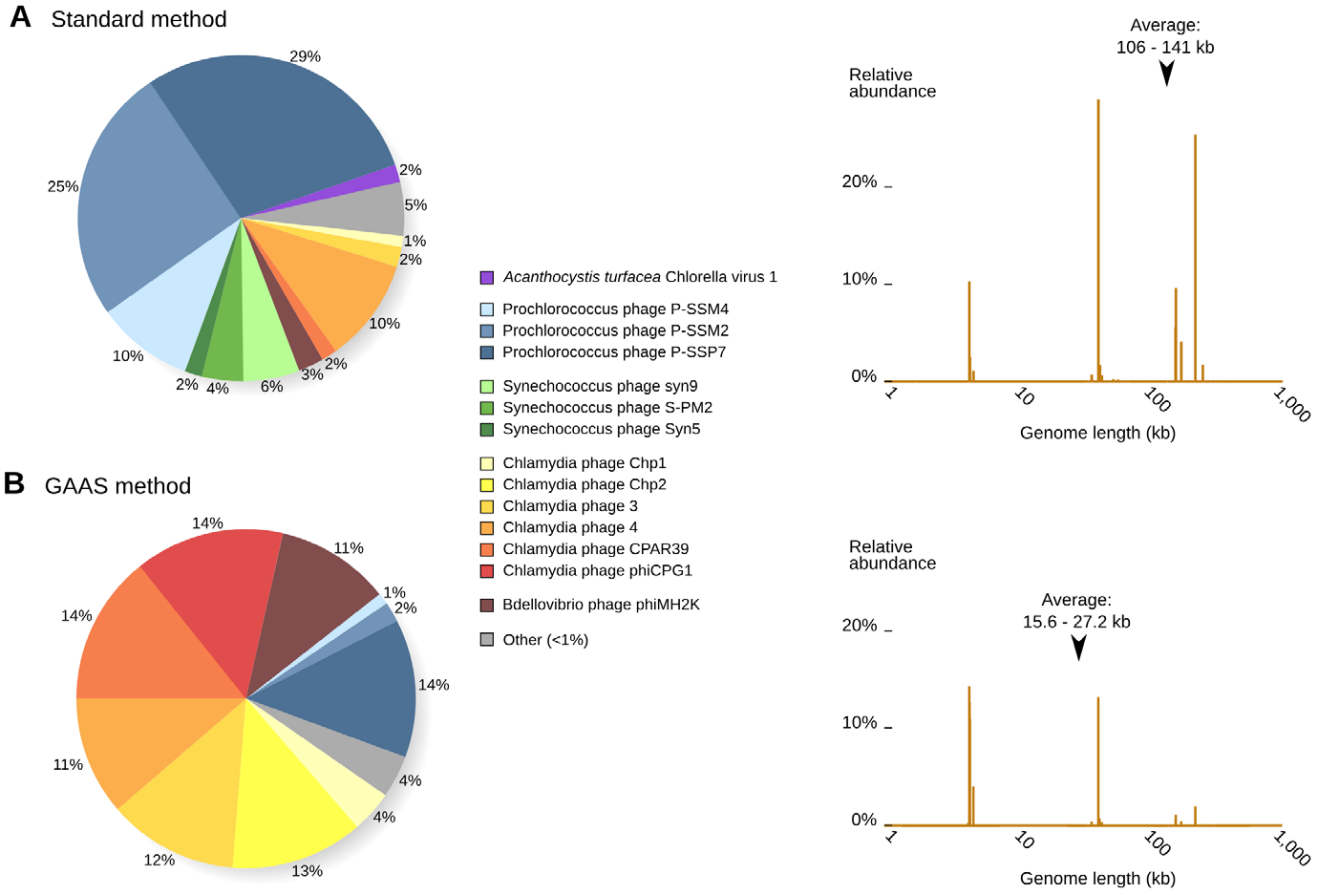
Re-analysis of the Sargasso Sea viral community. Genome relative abundance in the Sargasso Sea (left) and size spectrum with 95% confidence interval for the average genome length (right) were calculated using the standard method (A) and GAAS (B).

Re-analysis of the Sargasso Sea virome using GAAS revealed that small ssDNA phages were more important than previously assessed, representing ∼80% of the viral community (Figure 3). Community composition and average genome size for the Sargasso Sea virome were calculated using both the GAAS method and the standard method (no length normalization, top similarities only) for comparison. Both the pie charts and length spectra in Figure 3 were generated directly by GAAS. Using the standard method, the Sargasso Sea viral community was dominated by *Prochlorococcus* phages (64%), with lesser abundances of *Chlamydia* phages (15%), *Synechococcus* phages (12%), *Bdellovibrio* phages (3%) and *Acanthocystis chlorella* viruses (2%). In contrast, using GAAS, *Chlamydia* phages were the most abundant organism (79%), whereas *Prochlorococcus* phages only comprised 16% of the community. The presence of *Chlamydia* phages in the Sargasso Sea was previously verified experimentally using molecular methods [1]. In contrast to the standard method, the GAAS method also indicated very low relative abundances (<1%) of *Synechococcus* phages and *Chlorella* viruses, which have larger genomes.

Most of the variations in community composition estimates were explained by differences in viral genome lengths (Figure 3, right panel). The corrected relative abundance estimates provided by GAAS indicated that species with larger genomes were less abundant than previously thought, and that normalizing by genome length was essential for accurate estimation of community composition (as shown in benchmark tests, Figure 1). A lack of normalization could lead to poor and possibly misleading community composition estimates, as our results have shown, since relative abundance does not equal percentage of similarities.

Phages with small genomes (20–40 kb) are believed to be the most abundant oceanic viruses [11]. In the re-analysis of the Sargasso Sea metagenome, GAAS estimated that 80% of the viral particles were *Microviridae* (mainly *Chlamydia* phages), viruses with a genome size smaller than 10 kb. Multiple Displacement Amplification (MDA) was used during the preparation of the Sargasso Sea virome and could have led to over-representation of this viral family. Despite this potential bias, the *Chlamydia* phage content of this virome was still higher than in all viromes prepared with MDA (except for the stromatolite viromes [6]) (data not shown). In addition, diverse marine circovirus-like genomes, with a length of less than 3 kb, have also been reported in the Sargasso Sea [22], suggesting that small single-stranded viruses play important roles in this marine habitat.

### Average genome length varies significantly between and within biomes

**Figure 4:**
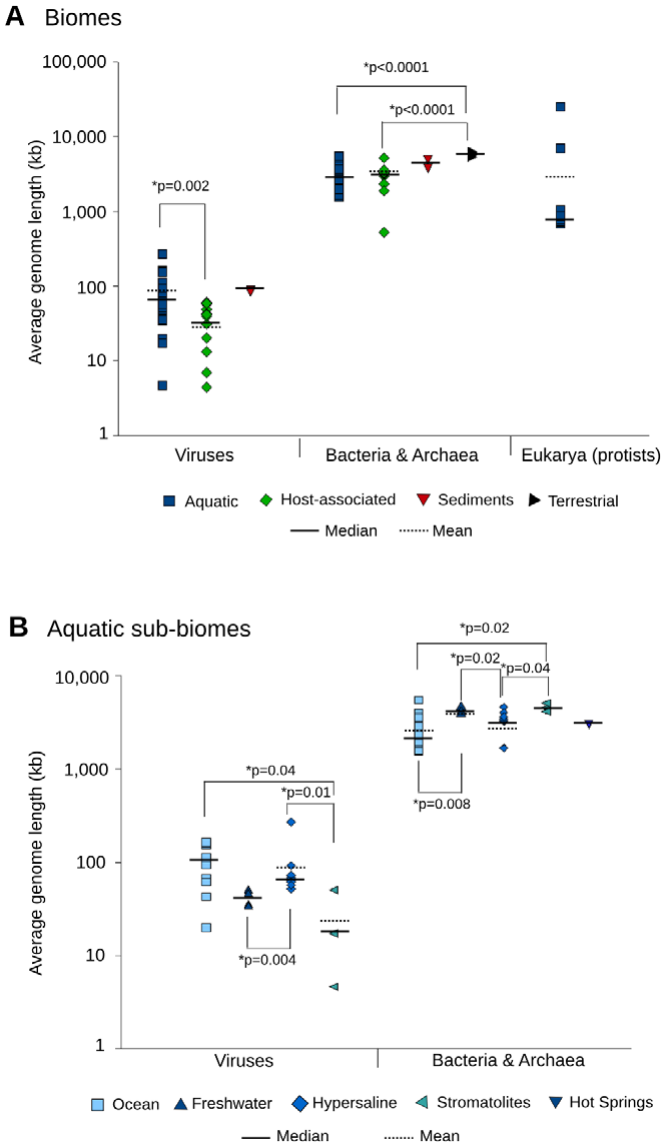
Average genome length of viruses, Bacteria and Archaea, and protists in metagenomes. Different biomes (A) and marine sub-biomes (B) were analyzed using GAAS. Non-parametric Mann-Whitney U tests were used to compare biomes. Metagenomes from sediments and hot springs were excluded from the statistical analysis due their small number. All protist metagenomes were from the ocean and could not be sub-classified further.

Both microbial and viral average genome lengths calculated by GAAS were significantly different between marine, terrestrial, and host-associated biomes (Figure 4A, Table S1, Table S2). Of the 169 metagenomes analyzed, 146 had a sufficient number of similarities for estimation of average genome length. The average for genome length across all aquatic viral metagenomes was consistent with the previous estimate of 50 kb for marine systems using PFGE by Steward et al. [9]. Host-associated and aquatic viromes had average genome lengths spanning a wide range, from 4.4 to 51.2 kb and from 4.6 to 267.9 kb respectively. Viral average genome lengths were significantly smaller in host-associated metagenomes than in aquatic systems (p = 0.002, Mann-Whitney U test). Estimates of microbial average genome length for aquatic and terrestrial biomes were similar to those predicted using the Effective Genome Size (EGS) method [15], a computational technique based on finding conserved bacterial and archaeal markers in metagenomic sequences. Aquatic microbiomes also showed large variation in average genome sizes, ranging from 1.5 to 5.5 Mb for Bacteria and Archaea and from 0.7 to 25.7 Mb for protists. Microbial average genome lengths in the terrestrial biome were significantly higher than in the host-associated and aquatic biomes (p<0.0001, Mann-Whitney U test). Genome lengths of Bacteria and Archaea from soil environments have previously been shown to be larger than those observed in other biomes [15]. A larger genome is characteristic of the copiotroph lifestyle [23] as it provides microbes a selective advantage in the complex soil environment where scarce but diverse resources are available [24].

Microbial and viral average genome lengths were also significantly different between aquatic sub-biomes. Aquatic metagenomes were grouped into five categories (ocean, freshwater, hypersaline, microbialites, and hot springs) to determine if the variation in average genome lengths could be accounted for by the influence of distinct sub-biomes (Figure 4B, Table S1, Table S2). Other biomes did not include enough metagenomes from different sub-biomes to allow for meaningful classification and analysis. While average genome lengths still varied over a range of values in sub-biomes, the variability was much lower than in the aquatic biome as a whole (Table S1). The average genome sizes in oceanic viromes varied from 20 to 163 kb, well within the range described in [17]. In hypersaline metagenomes, the average genome length varied from 51 to 263 kb, which is comparable to viral genome sizes detected in ponds of similar salinities [16]. A number of average genome lengths were significantly different between sub-biomes for both viruses and microbes (Figure 4B). The stromatolite metagenomes had an average genome length which was significantly different from the oceanic and hypersaline sub-biomes (p<0.05, Mann-Whitney U test), but not from freshwater systems. Oceanic and hypersaline environments were not significantly different. In comparison with the biome level (Figure 4A), the range of average genome lengths at the sub-biome level was reduced (Figure 4B). This suggests that differences in average genome lengths may be driven by environmental factors at a more specific level (e.g. the sub-biome) than what can be encompassed by general biome classifications. Previous work has demonstrated that both metabolic profiles and dinucleotide composition vary at the sub-biome level, and significant differences between both composition and metabolic functions have been reported for marine (ocean), hypersaline, microbialite, and freshwater environments [7],[25].

### Microbial and viral average genome lengths are independent

**Figure 5:**
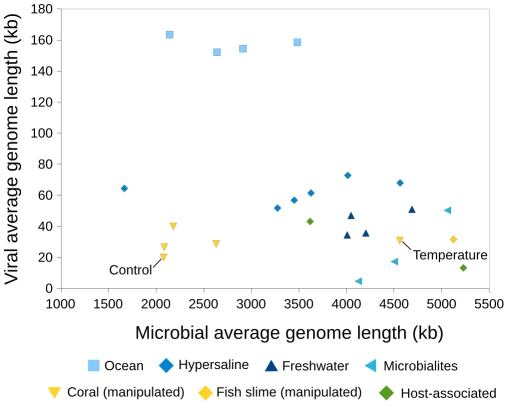
Relationship between average microbial and viral genome lengths in paired metagenomes.

Microbial and viral average genome lengths varied independently of each other across biomes and aquatic sub-biomes, and reflected differences in the way microbial and viral consortia react to stressors and environmental conditions (Figure 5). Using GAAS estimates for average genome lengths, we compared 25 pairs of viral and microbial metagenomes sampled from the same environment at the same time point. Viral and microbial community compositions have been shown previously to co-vary [26], however, there was no consistent trend between microbial and viral average genome length across all biomes (Kendall's tau = −0.21, p = 0.10).

Most viromes in this analysis were obtained by the collection of viral particles small enough to pass through 0.22 µm pore size filters. The four viral metagenomes collected using 0.45 µm filters [27] had a larger viral average genome length (in light blue in Figure 5). These data show that large viruses may be omitted when sampling with 0.22 µm filters and the capsid size of DNA viruses is likely positively correlated with their genome length. Sampling biases, however, do not account for the independence of viral and microbial length reported here.

Paired metagenomes from oceanic and hypersaline aquatic sub-biomes were characterized by small fluctuations in viral genome lengths coupled with large variations in microbial genome lengths. The four paired ocean metagenomes (Figure 5, light blue squares) were taken from waters surrounding coral atolls in the Northern Line Islands [27]. Microbial communities changed dramatically along a gradient of human disturbance, with populations of pathogens and heterotrophic microbes increasing with human activity [27], which could have resulted in large differences in average microbial genome lengths between atolls. Across all four atolls, viral communities were dynamic but dominated in general by *Synechococcus* and *Prochlorococcus* phage, according to both the original [27] and the GAAS analysis (not shown). The large genome of these widespread phages resulted in a less variable viral average genome length. In hypersaline metagenomes (Figure 5, blue diamonds), a similar trend of low variation in viral genome lengths coupled with larger ranges of microbial genome lengths was observed. This corresponded to known differences in the ranges of genome lengths of dominant halophilic viruses and microbes. The most abundant viruses in hypersaline systems have genome lengths between 32 and 63 kb, while predominant Halobacteria have genome lengths varying across a larger range, from 2.6 to 4.3 Mb [28],[29].

The relationship between viral and microbial average genome lengths in manipulated coral metagenomes reflected differences in how viral and microbial consortia reacted to stress (Figure 5, yellow triangles). Five of the six manipulated metagenome pairs used in this analysis were metagenomes from *Porites compressa* corals subjected to a variety of stressors [30],[31]. Nutrient, DOC, temperature, and pH stress all resulted in an increased abundance of large herpes-like viruses over the control, which could lead to increased average viral genome lengths overall [30]. However, shifts in the microbial consortia (consisting of Bacteria, Archaea, and eukaryotes) were more variable depending on which stressor was applied [31]. For example, temperature stressed corals showed a dramatic increase in fungal taxa, which could be driving the larger average microbial genome length seen here.

### Conclusions

The GAAS software package implements a novel methodology to accurately estimate community composition and average genome length from metagenomes with statistical confidence. GAAS provides the user with both textual and graphical outputs, including genome length spectra, relative abundance pie charts, and relative abundances mapped to phylogenetic trees. GAAS can easily be applied to any database of complete sequences to perform taxonomic or functional annotations, and provides filtering by relative alignment length as a standard for selecting significant similarities regardless of which database is used. Since GAAS controls for sampling bias towards larger genomes and considers all significant BLAST similarities, it has the potential to identify key players in ecosystems that may be ignored by other analyses. For example, the re-analysis of the Sargasso Sea virome indicated that small ssDNA phage were very abundant and may play a previously overlooked role in the oceanic ecosystem. GAAS could also be applied in metagenomic studies of disease outbreaks and epidemics. Many emerging and highly virulent human pathogens are ssRNA viruses with small genomes, which could be missed by standard analysis methods, which do not normalize for genome length. Meta-analysis using GAAS provided insight into how environmental factors may affect average genome lengths in microbial and viral communities and the relationships between them. The lack of covariance between microbial and viral average genome lengths indicates that natural and applied stressors have different effects on microbes and viruses from the same environment.

## Materials and Methods

### GAAS: Genome relative Abundance and Average Size in random shotgun libraries

#### GAAS software package

**Figure 6:**
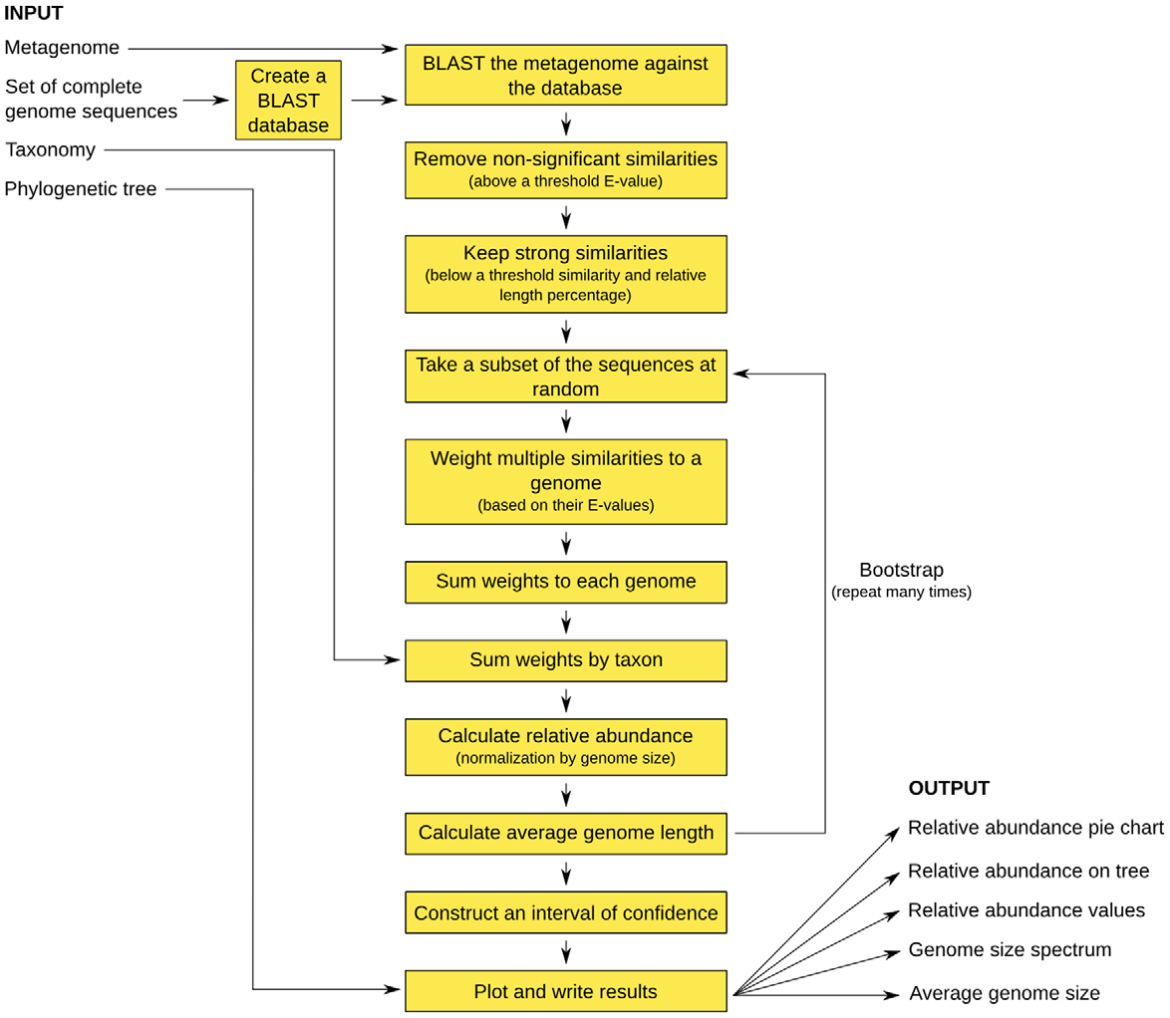
Flowchart of GAAS to calculate relative abundance and average genome size. GAAS runs BLAST and uses various corrections to obtain accurate estimations.

GAAS was implemented as a standalone software package in Perl and is freely available at http://sourceforge.net/projects/gaas/. It accepts and produces files in standard formats (FASTA sequences, Newick trees, tabular BLAST results, SVG graphics). The GAAS methodology is described in detail below and is outlined in Figure 6.

#### Similarity filtering

BLAST analyses (NCBI BLAST 2.2.1) were conducted through GAAS in order to determine significant similarities between metagenomic sequences and completely sequenced genomes. Similarities were filtered based on a combination of maximum E-value, minimum similarity percentage and minimum relative alignment length. E-value filtering removed non-significant similarities, and the alignment similarity percentage and relative length were used to select for strong similarities likely to reflect the taxonomy of the metagenomic sequences. E-values depend on the size of the database and the absolute length of alignments between query and target sequences, and thus may not be comparable between analyses [32],[33]. Relative alignment length, also called alignment coverage [34], is the ratio of the length of the alignment to the length of the query sequence (Figure S7). It is independent of the database size and sequence length, and provides an intuitive and consistent threshold to select significant similarities. Since the ends of sequenced reads can be of lower quality, similarities were kept only if the length of the alignment represented the majority of the length of the query sequence. Sequences with no similarity satisfying the filtering criteria were ignored in the rest of the analysis.

#### Similarity weighting

In order to avoid the loss of relevant similarities by reliance upon smallest E-values alone [5], all significant similarities for each query sequence (as defined by our criteria above) were kept and assigned weights as follows.

Based on the Karlin-Altschul equation, the expect value *E_ij_* between a metagenomic query sequence *i* and a target genome sequence *j* is given by: 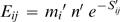 where *m'_i_* is the effective query sequence length, *n'* is the effective database size (in number of residues) and *S'_ij_* is the high-scoring pair (HSP) bitscore [32]. Using the effective length corrects for the “edge effect” of local alignment and is significant for sequences smaller than 200 bp such as sequences produced by the high throughput Roche-454 GS20 platform. Assuming that a query sequence is more likely to have local similarities to longer target genomes, each of the E-values can be reformulated into an expect value *F_ij_* of a similarity in a given target genome by: 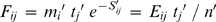 where *t'_j_* is the effective length [35] of the target genome *j*. Using the length of the target genome in the F-value produces an expect value relative to the target genome, not to the totality of the genome database (as is the case of the E-value).

From *F_ij_*, a weight *w_ij_* can be calculated as 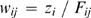 with *z_i_* being a constant such that for a given metagenomic query sequence *i*, 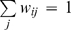. This weight carries the statistical meaning of the expect value of the similarity relative to the given genome in such a way that the larger the expect value, the lower the weight. Therefore, for a given query sequence *i*, the weight was calculated as 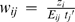.

#### Genome relative abundance using genome length normalization

The relative abundance of sequences in a random shotgun library is proportional not only to the relative abundance of the genomes in the library but also to their length. Similarly to the normalization used in proteomics [36]–[38], normalization by genome length is needed to obtain correct relative abundance of the species in a metagenome. For each target genome j, the weights w_ij_ to that genome were added to obtain W_j_. The weighted similarities W_j_ to each genome were then normalized by the actual length t_j_ of the genome (including chromosomes, organelles, plasmids and other replicons) to obtain accurate relative abundance estimates: 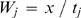 where x is a constant such that 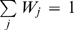.

#### Average genome length calculation

GAAS relies on the relatively stable genome size found within taxa [39] to calculate average genome length. The average genome length was calculated as a weighted average of individual genome lengths. The length of the genome for each individual organism identified in the metagenome was weighted by the relative abundance of that organism as calculated by GAAS. Thus, the mean genome length L was calculated as: 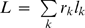 where r_j_ was the relative abundance of organism k, and l_j_ its individual genome length.

#### Confidence intervals for relative abundance and average genome length estimates

A bootstrap procedure was implemented in GAAS to provide empirical confidence intervals for relative abundance and average genome length estimates. The estimation of community composition and average genome length was repeated many times using a random subsample of 10,000 sequences for each repetition. Confidence intervals were determined based on the percentiles of the observed estimates, e.g. 5^th^ and 95^th^ percentiles for a 90% confidence interval.

### Reference databases for viral, microbial and eukaryotic metagenomes

NCBI RefSeq (ftp://ftp.ncbi.nih.gov/refseq/release) (Release 32, August 31, 2008) was used as the target database for the estimation of taxonomic composition and average genome size. Three databases containing exclusively complete genomic sequences were created from the viral, microbial, and eukaryotic RefSeq files. All incomplete sequences were identified as having descriptions containing words such as “shotgun”, “contig”, “partial”, “end” and “part”, and were removed from the database.

A taxonomy file containing only the taxonomic ID of the sequences in these three databases was produced using the NCBI Taxonomy classification. Sequences with a description matching the following words were excluded from that file unless the chromosomal sequences were also available for the same organism: “plasmid”, “transposon”, “chloroplast”, “plastid”, “mitochondrion”, “apicoplast”, “macronuclear”, “cyanelle” and “kinetoplast”. The complete viral, microbial, and eukaryal sequence files with accompanying taxonomic IDs are available at http://biome.sdsu.edu/gaas/data/.

### Mapping to phylogenetic trees

Similarly to the Interactive Tree Of Life (ITOL) [40] and MetaMapper (http://scums.sdsu.edu/Mapper), GAAS is able to graph the relative abundance of viral, microbial or eukaryotic species on phylogenetic trees such as the Viral Proteomic Tree (VPT) or Tree Of Life (http://itol.embl.de). The Viral Proteomic Tree was constructed using the approach introduced in the Phage Proteomic Tree and extending it to the >3,000 viral sequences present in the NCBI RefSeq viral collection (Edwards, R. A.; unpublished data, 2009).

### Benchmark using simulated viral metagenomes

Simulated metagenomes were created to test the validity and accuracy of the GAAS approach using the free software program Grinder (http://sourceforge.net/projects/biogrinder), which was developed in conjunction with GAAS. Grinder creates metagenomes from genomes present in a user-supplied FASTA file. Users can simulate realistic metagenomes by setting Grinder options such as community structure, read length and sequencing error rate. Over 9,500 simulated metagenomes based on the NCBI RefSeq virus collection were generated using Grinder. The viral database was chosen since its large amount of mosaicism and horizontal gene transfer represents a worst-case scenario. Therefore, benchmark results using the viral database are expected to be valid for higher-order organisms such as Bacteria, Archaea and eukaryotes. The parameters used were a coverage of 0.5 fold, and a sequencing error rate of 1% (0.9% substitutions, 0.1% indels). Half of the simulated metagenomes had a uniform rank-abundance distribution, while the other half followed a power law with model parameter 1.2. Sequence length in the artificial metagenomes was varied from 50 to 800 bp for the analysis of read length effects on GAAS estimates.

For each simulated viral metagenome, GAAS was run repeatedly with different parameter sets (relative alignment length and percentage of identity). The maximum E-value was fixed to 0.001 in order to remove similarities due to chance alone. Each set of variable parameters was tested on a minimum of 1,200 different Grinder-generated metagenomes. All computations were run on an 8-node Intel dual-core Linux cluster.

Due to the limited number of whole genome sequences available, a great majority of the sampled organisms in a metagenome cannot be assigned to a taxonomy. To evaluate the effect of sequences from novel organisms on GAAS estimates, the taxonomy of 80% randomly chosen organisms in the database was made inaccessible to GAAS rendering them “unknown”. A control simulation with 100% known organisms was run for comparison (Figure S2).

The accuracy of GAAS estimates was evaluated by comparing GAAS results to actual community composition and average genome size of the simulated metagenomes. The relative error for average genome size was calculated as 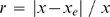, where *x* and *x_e_* are the true and estimated values respectively. For the composition, the cumulative error was calculated as 
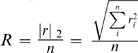
, where *r_i_* is the relative error on the relative abundance of the target genome *i* and *n* is the total number of sequences in the database.

Because the benchmark results were not normal, non-parametric statistical tests were used for all pairwise (Mann-Whitney U test) and multi-factor comparisons (Friedman test) of average errors. Non-parametric correlations were calculated using Kendall's tau.

### Benchmark using simulated microbial metagenomes

GAAS was also tested on the three simulated metagenomes available at IMG/m (http://fames.jgi-psf.org). Parameter setting and data processing were conducted as in viral benchmark experiments. Points on the IMG/m microbial benchmark graphs represent the average of 58 repetitions.

Microbial strains typically have a largely identical genome, with a fraction coding for additional genes and accounting for differences in genome length. An additional simulation was performed to investigate how the presence of closely related genomes influences the accuracy of the GAAS estimates. The 15 *Escherichia coli* strains present in the NCBI RefSeq database, ranging from 4.64 to 5.57 Mb in genome size, were used to produce ∼4,500 shotgun libraries with Grinder. The parameters used were the same as for the simulated viral metagenomes, but with a coverage of 0.0014 fold (>1,000 sequences). Half of the simulated metagenomes were treated as in the viral benchmark, using the GAAS approach and assuming no unknown species. The other half were treated similarly but taking only the top similarity. Points on the graph of the microbial strain benchmark represent the average of >2,200 repetitions.

### Meta-analysis of 169 metagenomes

The composition and average genome size for 169 metagenomes were calculated using GAAS. Most of these metagenomes were publicly available from the CAMERA [41], NCBI [42], or MG-RAST [43] (Table S2), and a few dozens were viromes and microbiomes newly collected from solar saltern ponds, chicken guts, different soils and an oceanic oxygen minimum zone (Protocol S1). The metagenomes used here therefore represent viral, bacterial, archaeal, and protist communities sampled from a diverse array of biomes and were categorized as one of the following: “aquatic”, “terrestrial”, “sediment”, “host-associated”, and “manipulated / perturbed”. The large number of aquatic metagenomes was further subdivided into: “ocean”, “hypersaline”, “freshwater”, “hot spring” and “microbialites”. Sampling, filtering, processing and sequencing methods differed among compiled metagenomes. Table 1 provides a summary of the number of metagenomes from each biome (a list of the complete dataset is presented in detail in Table S2).

**Table 1:** Summary of metagenomes by type used in the meta-analysis.

Biome	Sub-biome	Number of viral metagenomes	Number of bacterial and archaeal metagenomes	Number of protist metagenomes
Aquatic (total)	-	34	45	17
Aquatic	Ocean	15	26	17
Aquatic	Hypersaline	10	10	0
Aquatic	Freshwater	4	4	0
Aquatic	Hot spring	2	2	0
Aquatic	Microbialites	3	3	0
Sediments	-	3	2	0
Terrestrial (soil)	-	4	19	2
Host-associated	-	17	11	0
Manipulated / perturbed	-	7	8*	0

*: The five manipulated coral metagenomes also contained sequences from eukaryotic genomes as described in [31].

For all metagenomes, GAAS was run using a threshold E-value of 0.001, and an alignment relative length of 60%. In addition, for bacterial, archaeal and eukaryotic metagenomes, similarities were calculated using BLASTN with an alignment similarity of 80%. Due to the low number of similarities in viral metagenomes using BLASTN, TBLASTX was used for viruses, with a threshold alignment similarity of 75%. All average genome length estimates produced from less than 100 similarities were discarded to keep results as accurate as possible. Manipulated metagenomes were ultimately not used in the meta-analysis because they do not accurately represent environmental conditions. Statistical pairwise differences between average genome lengths across biomes were assessed using Mann-Whitney U rank-sum tests.

The average genome length and relative abundance results obtained for all metagenomes with our GAAS method were compared to the “standard” analytical approach where: 1) only the top similarity for each metagenomic sequence is kept, 2) there is no filtering by alignment similarity or relative length, and 3) no normalization by genome length is carried out. The virome from the Sargasso Sea was chosen to illustrate in detail the difference between the results obtained with the two methods (Figure 3).

### Correlation between viral and microbial average genome length

Average genome lengths were calculated for 25 pairs of microbial and viral metagenomes sampled from the same location at the same time. The statistical relationship between viral and microbial average genome length in paired metagenomes was evaluated using Kendall's tau, since lengths were not normally distributed. Regression analysis was performed with Generalized Linear Models (GLM). Interactions between genome lengths and biome classifications were not significant and were not included in final models.

### Statistical analyses

All statistical analyses of the GAAS benchmark results, environmental genome length and genome length correlations described above were performed using the free statistical software package R (http://www.R-project.org/) [44].

## Supporting Information

Protocol S1 Sample collection and metagenome sequencing
(0.32 MB PDF)

Table S1 Biome averaged genome length estimated by GAAS for the metagenomes of each environment. The numbers reported are: mean (median) ± standard deviation.
(0.22 MB PDF)

Table S2 Detail of the 169 metagenomes used for the meta-analysis and their average genome size estimated by GAAS. Accession numbers: CA, CAMERA Accession; GB, NCBI GenBank; GP, NCBI Genome Project; GSS, NCBI Genome Survey Sequence; MG: MG-RAST Accession; SRA, NCBI Short Read Archive.
(0.24 MB PDF)

Figure S1 Sampling bias toward larger genomes in metagenomic libraries. Larger genomes will produce more fragments of a given size, and are more likely to be sampled even if they occur in the same abundance as small genomes.
(0.17 MB TIF)

Figure S2 Accuracy of the GAAS estimates when no species are unknown. Error on the relative abundance (top) and average genome size estimates (bottom) when: (A) 80% of the species were treated as unknown, (B) no species were assumed to be unknown. The simulated viromes were made of 100 bp sequences.
(0.29 MB TIF)

Figure S3 Accuracy of GAAS estimates for microbial metagenomes. GAAS relative abundance error (top), average genome size error (middle) and number of similarities (bottom) for the JGI simulated microbial metagenomes (∼1,200 bp/read). 80% of the species were treated as unknown.
(0.39 MB TIF)

Figure S4 Effect of using all similarities for microbial strains. The error on community composition (top) and average genome length (bottom) for simulated metagenomes made of 15 Escherichia coli strains was estimated by GAAS. Sequence length was 100 bp and no strains were treated as unknown.
(0.27 MB TIF)

Figure S5 Effect of metagenomic sequence length on the accuracy of GAAS estimates. Error was calculated for the relative abundance (top) and average genome length (bottom) estimates. 80% of the species in the viral simulated metagenomes were treated as unknown.
(0.64 MB TIF)

Figure S6 Error surfaces for Figure S5. The two surfaces of each graph correspond to the average error ± the standard deviation for the >1,200 simulated metagenomes.
(0.62 MB TIF)

Figure S7 The relative alignment length filtering parameter. The relative alignment length is defined as the ratio of the length of the alignment over the length of the query sequence length, expressed in percent.
(0.14 MB TIF)
