# Supplementary material for: The GAAS Metagenomic Tool and Its Estimations of Viral and Microbial Average Genome Size in Four Major Biomes
Source: PLoS Comput Biol. 2009 Dec 11;5(12):e1000593. doi: 10.1371/journal.pcbi.1000593 (PMC2781106; doi:10.1371/journal.pcbi.1000593)
Supplement: Protocol S1 — Sample collection and metagenome sequencing (0.32 MB PDF) [file pcbi.1000593.s001.pdf]

Protocol S1: Sample collection and metagenome sequencing

Oxygen minimum zone viromes:

The oceanic oxygen minimum zone samples were collected in June 2008 off Iquique, Chile, (20.104° S and 70.404° W). Oxygen minimum zone viral metagenomes were constructed by filtering 40 l of water collected using a CTD rosette lowered to a sampling depth of 90 and 200 m (OxMinZoneVir200806-90 and OxMinZoneVir200806-200 respectively). Samples were concentrated through a 100 kDa tangential flow filter to retain viral particles. The concentrate was passed through a 0.45 μm sterivex filter to remove larger cells and treated with chloroform. The viruses were purified using cesium chloride (CsCl) step gradients to remove free DNA and any cellular material. Viral samples were visually checked for microbial contamination using epifluorescence microscopy. Viral DNA was extracted using CTAB/phenol:chloroform extractions and amplified using Genomiphi reactions. These reactions were pooled and purified using silica columns (Qiagen Inc, Valencia, CA). The DNA was precipitated with ethanol and re-suspended in water at a concentration of approximately 300 ng μl^{-1}. Sequencing was performed using pyrosequencing on Roche Applied Sciences/454 Life Sciences GS-FLX platforms with a practical limit of 250 bp. Duplicate sequences were removed from the obtained dataset and the submitted to NCBI (Genome Project 40791 and 40793).

Runting-stunting chicken gut viromes:

One day old specific pathogen-free broilers (USDA-ARS, SEPRL, Athens, GA) were orally infected with 1 ml of gut content from 12-day-old commercial broiler chickens which showed the typical signs of runting-stunting-syndrome (RSS) in chicken (growth retardation > 40%, cystic lesions

in the small intestine). Before inoculation, the gut content of RSS affected chicken was centrifuged at 4°C for 30 min at 3000 x g. the obtained supernatant was filtered first through a 0.45 μm filter followed by filtration through a 0.22 um filter. A second group of broilers was mock-infected with phosphate buffered saline. Five days, 8 d, and 12 d after infection, 10 birds of each group were euthanized and necropsy was performed. The duodenal loop was taken for histological examination. The analysis of the sections showed that cystic lesions were only present in the infected group. The highest number of lesions was observed at 8 d after infection. Based on this result the gut content harvested at 5 d after infection was used for subsequent experiments. The purification of the gut content was performed following a multi-step centrifugation protocol. In a first step, the samples were centrifuged at 16000 x g to remove cellular organelles and debris. The obtained supernatant was filtered twice as described above. Next the filtrate was centrifuged through a 10% sucrose cushion made in TEN buffer (10 mM Tris-HCl, 100 mM NaCl, 1 mM EDTA, pH 7.5) at 174899 x g for 3h. The obtained pellets (RSS+, RSS-) were resuspended in 400 μl TEN buffer. To purify nucleic acids, the RNA and DNA localized outside of viral particles needed to be degraded. To this end, 40 μl of 10x DNA I buffer (Roche), 20 units of DNase I (Roche) and 10 ug of RNase I (Roche) was added to 360 μl of the viral suspension. The mixture was incubated for 1 h at 37C. Both samples (RSS+, Con) were then split and 200 μl of each sample was used for purification of either the DNA (QIAamp DNA Blood Mini Kit, Qiagen) or RNA (High Pure RNA isolation Kit, Roche) following the manufacturers instructions. The resulting samples (RSS+ RNA, RSS- RNA, RSS+ DNA and RSS- DNA) were amplified separately using two different protocols to amplify the metagenome (called respectively ChickenRuntingStuntingPRnaVir2008, ChickenRuntingStuntingMRnaVir2008, ChickenRuntingStuntingPDnaVir2008 and ChickenRuntingStuntingMDnaVir2008). The RNA

containing samples were amplified using the Transplex Whole Transcriptome Amplification Kit (Sigma). The amplification of the DNA library for both DNA samples was performed using GenomiPhi V2 DNA Amplification Kit (GE Healthcare). Both protocols were applied as recommended by the manufacturer. The resulting cDNA library was submitted to 454 Life Science for sequencing using the GS-FLX platform. Duplicate sequences were removed and submitted to NCBI (Genome Project 40789, 40785, 40787 and 40783).

Solar saltern microbiome:

A water sample from the solar saltern of South Bay Salt Works (Chula Vista, CA) was collected in July 2004 from a pond with high salinity (28-30%, measured using a hand refractometer). The microbial fraction was isolated from the water sample by passage through a 0.2 μm tangential flow filter (TFF, Millipore). The retentate was kept and the microbial fraction was collected from the 0.2 μm TFF retentate by centrifugation at ~ 2000 xg for 10 min. Microbial DNA was extracted using the Ultra Clean Soil DNA Kit (Mo Bio Laboratories, CA). The microbial DNA samples was amplified using the strand-displacement Φ29 DNA polymerase (GenomiPhi Amersham Biosciences, NJ). The resulting metagenomic DNA was pyrosequenced on the GS20 sequencer (454 Life Sciences, CT). The raw metagenomic sequences were screened to remove duplicate sequences. The metagenome, referred to as HighSalternSDbayMicD200407, was submitted to NCBI (Genome Project 40795).

South China sediments microbiome:

A marine sediment sample was collected using a gravity piston corer during a March 2006

Marine Expedition at the BD7-2 station of the South China Sea at a depth of 778.5 m below seafloor. The sample was stored onboard at 4°C and then divided into 5-cm sediment subsamples below seafloor. and stored in −80°C. The 5 to 10 cm layer was used for the library construction in this study. Prior to the metagenome DNA extraction, marine sediments were washed following the protocol previously described by Fortin [1] to remove contaminants: three washes, each wash with 100ml washing buffer (50mM Tris-HCl, pH 9.0, 100mM Na2EDTA, 1.0% PVP, 100mM NaCl, 0.05% Triton X-100), after vortexing for 1 min, the sample was incubated in 55°C for 3 min, and then centrifuged at 3,000×g for 5 min [1]. After washing steps, 5g pellet was mixed by vortexing with 13.5 ml of extraction buffer (100 mM Tris-HCl, pH 8.0, 100 mM sodium EDTA, pH 8.0, 100 mM sodium phosphate, pH 8.0, 1.5 M NaCl, 1% CTAB). Three cycles of thawing and freezing in liquid nitrogen were then applied to the suspension and the sample was then incubated at 37°C with 50 μl of proteinase K (20 mg/ml) for 30 min [2,3]. The extracted metagenomic DNA was repaired using Epicentre's repair enzyme mix and size-selected on 1% agarose PFGE with CHEF-DRIII system (Bio-Rad). Pulsed-field gel electrophoresis was carried out at 5 V/cm voltages with a ramping time of 0.1s to 40s at 14°C in 0.5×TBE buffer for 16 h. The metagenomic DNA with size of 36 to 48 kb was cut off from the gel and recovered by electro elution and then ligated to Epicentre's pCC2 FOS fosmid vector. This metagenomic library, named SouthChinaSeaSedimentsMic, was constructed using Epicentre' CopyControl fosmid library production kit. Over 1000 fosmid clones were randomly selected from the IMCAS-F003 library for end sequencing using T7 primer (5'-TAATACGACTCACTATAGGG-3') and pCC2 reverse sequencing primer (5'-CAGGAAACAGCCTAGGAA-3'). All the fosmid end sequences were revised and trimmed using Lasergene package, version 7.10 (DNA star, USA) before submission to NCBI (Genome Project 33581).

Pacific Beach sand metagenome:

DNA was extracted from a sample of sand at Pacific Beach, San Diego, California, USA, in august 1999, cloned and sequenced. The protocol was described in detail by Naviaux [4]. Here, over 2,300 additional clones from this metagenomic library, named PacificBeachSandEuk here, were sequenced following the same procedure as before and the full set of sequences (~4900) was made publicly available through the NCBI (Genome Project 13729).

Fish gut viromes:

Adult hybrid striped bass were collected in April 2006 from a 5x2 m open-air aquaculture pond in San Diego, California, USA. Each fish was classified as healthy or morbid by veterinarians upon visual inspection. Fish were sacrificed with an overdose of MS-222 (Finquel, Argent Laboratories), and examined for the presence of gross external and internal lesions to confirm preliminary diagnoses. Symptomatic fish had empty gut contents. Five symptomatic and five asymptomatic fish were selected and gut contents were collected by flushing aseptically with 10 mL of SM buffer. Samples were sonicated (15 seconds, 3 times) and then centrifuged at 150 x g for 20 minutes at 4°C. The supernatant was then filtered (0.45 μm and 0.2 μm) to separate the microbial fraction (attached onto the filter) from the viruses (filtrate). Viral particles in the filtrate were purified using a CsCl step gradient and viral DNA was extracted as described by Thurber [5]. Viral DNA was amplified with GenomiPhi (GE Healthcare, Piscataway, NJ) and ethanol precipitated. Approximately 10 μg of each DNA sample was submitted for GS20 pyrosequencing at 454 Life Sciences to produce the FishHealGutKentSTVir20060504 and FishMorGutKentSTVir20060504 metagenomes (from healthy fish and morbid fish respectively). Duplicate sequences were removed and the metagenomes were

submitted to NCBI (Genome Project 28397 and 28399).

Arctic marine microbial metagenome:

The Arctic sample (ArcticMic) was collected from a depth of 10 m at 72:19.33N, 151:59.07W [6]. Environmental DNA was extracted from the bacterial size fraction obtained by pumping 500 L of seawater sequentially through a 1 μm nominal pore size polypropylene string-wound filter (Cole Parmer) and a 0.8 μm polycarbonate filter (Nuclepore). Bacteria were collected from the filtrate by tangential flow filtration using a 0.1 μm hollow fiber filter (A/G Technology) and an Amicon DC10 gear pump. The sample was concentrated to 2 L, diafiltered with a buffer (0.5 M NaCl, 0.1 M EDTA, 10 mM Tris pH 8.0), and stored frozen. Cells were later collected from the thawed concentrate and lysed by treatment with SDS and lysozyme. Nucleic acids were extracted from the lysate using phenol and chloroform, sequenced, and released (Genome Project 29035).

Soil microbiomes:

Soil cores were taken to a depth of 5 cm from a random location in proximity of the land-use type associated with primary and secondary tower locations at selected National Ecological Observatory Network (NEON) primary sites. NEON soils were collected and stored at -20 °C. Prior to downstream analysis soil samples were passed through an 8 mm sieve in order to remove roots and any associated surface litter. After sieving, remaining fine roots were hand-picked from the soil with tweezers. Metagenomic DNA was isolated from 5-10 g of soil using the UltraClean® Mega Soil DNA Isolation Kit (MOBIO, Carlsbad, CA). Concentration and quality assessment was determined by

fluorometry (Qubit Quantitation Platform, Invitrogen, Carlsbad, CA) and agarose gel electrophoresis. Metagenomic DNA (5 ug) was used to construct shotgun libraries and prepared for sequencing using the standard GS FLX emPCR protocol and LR70 sequencing chemistry (Roche Applied Science, Indianapolis, IN). Sequencing was performed by the High-Throughput Genome Analysis Core (HGAC), Institute for Genomics and Systems Biology at Argonne National Laboratory. MG-RAST accession numbers are:

Metagenome	MG-RAST ID
SoilSJ1Mic	MG 4441557.3
SoilWF1Mic	MG 4441556.3
SoilHF1Mic	MG 4441642.3
SoilKP3Mic	MG 4441643.3
SoilLF2Mic	MG 4441644.3
SoilSJ2Mic	MG 4441645.3
SoilKW1Mic	MG 4441664.3
SoilWF2Mic	MG 4441665.3
SoilYN2Mic	MG 4441687.3
SoilTF1Mic	MG 4441688.3
SoilCP1Mic	MG 4441689.3
SoilCC1Mic	MG 4441690.3
SoilCP3Mic	MG 4441691.3
SoilKW2Mic	MG 4441691.4
SoilKP1Mic	MG 4441994.3
SoilTF2Mic	MG 4442452.3
SoilYN1Mic	MG 4442453.3
SoilLF1Mic	MG 4442455.3

Microbial metagenomes of the Indian Ocean and Antarctica lakes:

These metagenomes were collected during phase II of the Global Ocean Sampling effort [7,8] and during an Antarctica expedition. While these data is unpublished, the sequences and metadata for

these samples are available on CAMERA and NCBI:

Metagenome	NCBI Genome Project ID
AntarcticaLakeMic	GP 33179
GS000a11Mic	GP 13694
GS000a13Mic	GP 13694
GS000b11Mic	GP 13694
GS000b13Mic	GP 13694
GS000cMic	GP 13694
GS000dMic	GP 13694
GS001aEuk	GP 13694
GS001bEuk	GP 13694
GS011Mic	GP 13694
GS012Mic	GP 13694
GS016Mic	GP 13694
GS020Mic	GP 13694
GS023Mic	GP 13694
GS025Euk	GP 19735
GS034Mic	GP 13694
GS048aMic	GP 13694
GS048bEuk	GP 13694
GS108bEuk	GP 13694
GS110bEuk	GP 13694
GS112bEuk	GP 13694
GS117bEuk	GP 13694
GS122bEuk	GP 13694
Move858Vir	GP 13694

References:

1. Fortin N, Beaumier D, Lee K, Greer CW (2004) Soil washing improves the recovery of total community DNA from polluted and high organic content sediments. J Microbiol Methods 56: 181-191.

2. Kauffmann IM, Schmitt J, Schmid RD (2004) DNA isolation from soil samples for cloning in different hosts. Appl Microbiol Biotechnol 64: 665-670.

3. Zhou J, Bruns MA, Tiedje JM (1996) DNA recovery from soils of diverse composition. Appl Environ Microbiol 62: 316-322.

4. Naviaux RK, Good B, McPherson JD, Steffen DL, Markusic D, et al. (2005) Sand DNA - a genetic library of life at the water's edge. Mar Ecol Prog Ser 301: 9-22.

5. Thurber RV, Haynes M, Breitbart M, Wegley L, Rohwer F (2009) Laboratory procedures to generate viral metagenomes. Nat Protocols 4: 470-483.

6. Cottrell MT, Yu L, Kirchman DL (2005) Sequence and expression analyses of Cytophaga-like hydrolases in a Western Arctic metagenomic library and the Sargasso Sea. Appl Environ Microbiol 71: 8506-8513.

7. Venter JC, Remington K, Heidelberg JF, Halpern AL, Rusch D, et al. (2004) Environmental genome shotgun sequencing of the Sargasso Sea. Science 304: 66-74.

8. Rusch DB, Halpern AL, Sutton G, Heidelberg KB, Williamson S, et al. (2007) The Sorcerer II global ocean sampling expedition: northwest Atlantic through eastern tropical Pacific. PLoS Biol 5: e77.
