# Supplementary material for: The GAAS Metagenomic Tool and Its Estimations of Viral and Microbial Average Genome Size in Four Major Biomes
Source: PLoS Comput Biol. 2009 Dec 11;5(12):e1000593. doi: 10.1371/journal.pcbi.1000593 (PMC2781106; doi:10.1371/journal.pcbi.1000593)
Supplement: Table S1 — Biome averaged genome length estimated by GAAS for the metagenomes of each environment. The numbers reported are: mean (median) ± standard deviation. (0.22 MB PDF) [file pcbi.1000593.s002.pdf]

Table S1: Biome-averaged genome length estimated by GAAS for the metagenomes of each environment. The numbers reported are: mean (median) ± standard deviation

Biome	Sub-biome	Average viral genome length (kb)	Average bacterial and archaeal genome length (kb)	Average protist genome length (kb)
Aquatic (total)	-	79.9 (61.4) ± 59.4	3,020 (2,970) ± 1,150	2,690 (807) ± 6,060
Aquatic	Ocean	102 (103) ± 53.3	2,580 (2,150) ± 1,120	-
Aquatic	Hypersaline	91.9 (66.2) ± 72.2	3,250 (3,430) ± 925	-
Aquatic	Freshwater	42.0 (41.4) ± 8.23	4,240 (4,130) ± 313	-
Aquatic	Hot spring	-	2,820 (2,560) ± 1,340	-
Aquatic	Stromatolites	24.1 (17.3) ± 23.6	4,560 (4,500) ± 157	-
Sediments	-	85.6 (85.6) ± 4.28	4,370 (4,370) ± 803	-
Terrestrial (soil)	-	-	5,910 (5,930) ± 218	-
Host-associated	-	33.2 (39.8) ± 21.2	3,150 (3,190) ± 1,420	-
