# Supplementary material for: The GAAS Metagenomic Tool and Its Estimations of Viral and Microbial Average Genome Size in Four Major Biomes
Source: PLoS Comput Biol. 2009 Dec 11;5(12):e1000593. doi: 10.1371/journal.pcbi.1000593 (PMC2781106; doi:10.1371/journal.pcbi.1000593)
Supplement: Table S2 — Detail of the 169 metagenomes used for the meta-analysis and their average genome size estimated by GAAS. Accession numbers: CA, CAMERA Accession; GB, NCBI GenBank; GP, NCBI Genome Project; GSS, NCBI Genome Survey Sequence; MG: MG-RAST Accession; SRA, NCBI Short Read Archive. (0.24 MB PDF) [file pcbi.1000593.s003.pdf]

Table S2: Detail of the 169 metagenomes used for the meta-analysis and their average genome size estimated by GAAS. Accession numbers: CA, CAMERA Accession; GB, NCBI GenBank; GP, NCBI Genome Project; GSS, NCBI Genome Survey Sequence; MG: MG-RAST Accession; SRA, NCBI Short Read Archive.

Metagenome name	Type	Biome	Sub-biome	Microbial-viral pairing	Accession number	Reference	Est. avg. genome length (kb)
AlaskanSoilFungalEuk	eukaryal	Terrestrial	-	-	GP 28853	Allen et al., ISME J., 2009 [1]	-
AlicanteSalternMic	microbial	Aquatic	Hypersaline	-	GSS DU824018-DU826964	Legault et al., BMC Genomics 2006 [2]	3,190.26
AlvinellaWormMic	microbial	Host-associated	-	-	GP 17241	Grzymski et al., Proc. Nat. Acad. Sci. USA 2008 [3]	2,351.67
AntarcticaLakeMic	microbial	Aquatic	Hypersaline	-	GP 33179	-	1,657.26
ArcticMic	microbial	Aquatic	Ocean	Arctic Ocean	GP 29035	-	1,606.13
ArcticVir2002	viral	Aquatic	Ocean	Arctic Ocean	GP 17769	Angly et al., PLoS Biology 2006 [4]	66.67
BabyFecesSDVir	viral	Host-associated	-	-	GB ED651217-ED651693	Breitbart et al., Res. Microbiol. 2008 [5]	-
BBCVir1996to2004	viral	Aquatic	Ocean	-	GP 17767	Angly et al., PLoS Biology 2006 [4]	42.75
BearpawHotSpringVir	viral	Aquatic	Hot spring	-	GP 18929	Pride et al., BMC Genomics 2008 [6]	-
CheseapeakeBayVir	viral	Aquatic	Ocean	Chesapeake Bay	GP 16522	Bench et al., Appl. Environ. Microbiol. 2007 [7]	112.38
ChickenCecumCJejuniMic2007	microbial	Host-associated	-	Chicken Cecum	GP 28599	Qu A et al., PLoS One, 2008 [8]	3,695.38
ChickenCecumUninfectedMic2007	microbial	Host-associated	-	Chicken cecum	GP 28597	Qu A et al., PLoS One, 2008 [8]	-
ChickenRuntingStuntingMDnaVir2008	viral	Host-associated	-	Chicken cecum	GP 40783	-	39.77
ChickenRuntingStuntingMRnaVir2008	viral	Host-associated	-	Chicken cecum	GP 40785	-	41.99

ChickenRuntingStuntingPDnaVir2008	viral	Host-associated	-	Chicken cecum	GP 40787	-	31.10
ChickenRuntingStuntingPRnaVir2008	viral	Host-associated	-	Chicken cecum	GP 40789	-	6.93
ConPorCompHawMic200602	microbial	Manipulated / Perturbed	-	Control Pcomp	GP 28429	Vega Thurber et al., Env. Mic. 2009 [9]	2,077.79
ConPorCompHawVir200602	viral	Manipulated / Perturbed	-	Control Pcomp	GP 28417	Vega Thurber et al., Proc. Nat. Acad. Sci. USA 2008 [10]	19.90
DesertSoilJoshuaTreeVir	viral	Terrestrial	-	-	GSS ER781257-ER785833	Fierer et al., Appl. Environ. Microb. 2007 [11]	-
DOCPorCompHawMic200602	microbial	Manipulated / Perturbed	-	DOC Pcomp	GP 28433	Vega Thurber et al., Env. Mic. 2009 [9]	2,630.08
DOCPorCompVirHaw200602	viral	Manipulated / Perturbed	-	DOC Pcomp	GP 28421	Vega Thurber et al., Proc. Nat. Acad. Sci. USA 2008 [11]	28.61
FannLIMic20050811	microbial	Aquatic	Ocean	Fanning island	GP 28367	Dinsdale et al., PLoS One 2008 [12]	2,638.17
FannLIVir20050811	viral	Aquatic	Ocean	Fanning island	GP 28369	Dinsdale et al., PLoS One 2008 [12]	152.09
FishHealGutKentSTMic20060504	microbial	Host-associated	-	Fish gut	GP 28389	Dinsdale et al., Nature 2008 [13]	5,234.77
FishHealGutKentSTVir20060504	viral	Host-associated	-	Fish gut	GP 28397	-	-
FishHealSlimKentSTMic20060504	microbial	Host-associated	-	Fish slime	GP 28393	Dinsdale et al., Nature 2008 [13]	5,229.78
FishHealSlimKentSTVir20060504	viral	Host-associated	-	Fish slime	GP 28401	Dinsdale et al., Nature 2008 [13]	13.23
FishMorGutKentSTMic20060504	microbial	Manipulated / Perturbed	-	Fish Morbid Gut	GP 28391	Dinsdale et al., Nature 2008 [13]	5,260.79
FishMorGutKentSTVir20060504	viral	Manipulated / Perturbed	-	Fish Morbid Gut	GP 28399	-	-
FishMorSlimKentSTMic20060504	microbial	Manipulated / Perturbed	-	Fish Morbid Slime	GP 28395	Dinsdale et al., Nature 2008 [13]	5,126.12

FishMorSlimKentSTVir20060504	viral	Manipulated / Perturbed	-	Fish Morbid Slime	GP 28403	Dinsdale et al., Nature 2008 [13]	31.57
GOMVir1994to2001	viral	Aquatic	Ocean	Gulf of Mexico	GP 17765	Angly et al., PLoS Biology 2006 [4]	61.04
GS000a11Mic	microbial	Aquatic	Ocean	Sargasso Sea	GP 13694	Venter et al., Science 2004 [14]	5,465.57
GS000a13Mic	microbial	Aquatic	Ocean	Sargasso Sea	GP 13694	Venter et al., Science 2004 [14]	5,465.57
GS000b11Mic	microbial	Aquatic	Ocean	Sargasso Sea	GP 13694	Venter et al., Science 2004 [14]	1,948.84
GS000b13Mic	microbial	Aquatic	Ocean	Sargasso Sea	GP 13694	Venter et al., Science 2004 [14]	1,948.84
GS000cMic	microbial	Aquatic	Ocean	Sargasso Sea	GP 13694	Venter et al., Science 2004 [14]	2,292.36
GS000dMic	microbial	Aquatic	Ocean	Sargasso Sea	GP 13694	Venter et al., Science 2004 [14]	1,599.07
GS001aEuk	eukaryal	Aquatic	Ocean	-	GP 13694	Venter et al., [14] Science 2004	24,690.91
GS001bEuk	eukaryal	Aquatic	Ocean	-	GP 13694	Venter et al., Science 2004 [14]	6,921.47
GS011Mic	microbial	Aquatic	Ocean	-	GP 13694	Rush et al., PLoS Biol. 2007 [15]	1,543.51
GS012Mic	microbial	Aquatic	Ocean	Chesapeake Bay	GP 13694	Rush et al., PLoS Biol. 2007 [15]	1,658.76
GS016Mic	microbial	Aquatic	Ocean	Gulf of Mexico	GP 13694	Rush et al., PLoS Biol. 2007 [15]	1,564.53
GS020Mic	microbial	Aquatic	Ocean	-	GP 13694	Rush et al., PLoS Biol. 2007 [15]	2,896.19
GS023Mic	microbial	Aquatic	Ocean	-	GP 13694	Rush et al., PLoS Biol. 2007 [15]	1,671.19
GS025Euk	eukaryal	Aquatic	Ocean	-	GP 19735	Rush et al., PLoS Biol. 2007 [15]	895.31
GS034Mic	microbial	Aquatic	Ocean	-	GP 13694	Rush et al., PLoS Biol. 2007 [15]	2,002.14
GS048aMic	microbial	Aquatic	Ocean	-	GP 13694	Rush et al., PLoS Biol. 2007 [15]	1,718.17
GS048bEuk	eukaryal	Aquatic	Ocean	-	GP 13694	Rush et al., PLoS Biol. 2007 [15]	691.96
GS108bEuk	eukaryal	Aquatic	Ocean	-	GP 13694	-	686.97
GS110bEuk	eukaryal	Aquatic	Ocean	-	GP 13694	-	935.95
GS112bEuk	eukaryal	Aquatic	Ocean	-	GP 13694	-	782.20
GS117bEuk	eukaryal	Aquatic	Ocean	-	GP 13694	-	765.82

GS122bEuk	eukaryal	Aquatic	Ocean	-	GP 13694	-	719.78
GutlessWormMic	microbial	Host-associated	-	-	GB AASZ01000000	Woyke et al., Nature 2006 [16]	522.88
HBCStromBahamasMic20050111	microbial	Aquatic	Microbialites	Bahamas	GP 28383	Dinsdale et al., Nature 2008 [13]	4,125.02
HBCStromBahamasVir20050111	viral	Aquatic	Microbialites	Bahamas	GP 28381	Desnues et al., Nature 2008 [17]	4.63
HealSputRep3SDVir20060707	viral	Host-associated	-	-	GP 28439	Dinsdale et al., Nature 2008 [13]	-
HighSalternSDbayMic20051128	microbial	Aquatic	Hypersaline	High saltern SD 200511	GP 28453	Dinsdale et al., Nature 2008 [13]	3,407.29
HighSalternSDbayMicD200407	microbial	Aquatic	Hypersaline	High saltern SD	GP 40795	-	3,275.39
HighSalternSDbayVir20051116	viral	Aquatic	Hypersaline	High saltern SD	GP 28457	Dinsdale et al., Nature 2008 [13]	51.73
HighSalternSDbayVir20051128	viral	Aquatic	Hypersaline	High saltern SD 200511	GP 28451	Dinsdale et al., Nature 2008 [13]	267.95
HighSalternSDbayVir20051207	viral	Aquatic	Hypersaline	High saltern SD	GP 28447	Dinsdale et al., Nature 2008 [13]	-
Hot10Mic20021007	microbial	Aquatic	Ocean	-	GSS DU731018-DU796676, DU800850-DU800864	DeLong et al., Science 2006 [18]	1,838.16
Hot130Mic20021006	microbial	Aquatic	Ocean	-	GSS DU731018-DU796676, DU800850-DU800864	DeLong et al., Science 2006 [18]	3,849.65
Hot200Mic20021006	microbial	Aquatic	Ocean	-	GSS DU731018-DU796676, DU800850-DU800864	DeLong et al., Science 2006 [18]	1,868.80
Hot4000Mic20031221	microbial	Aquatic	Ocean	-	GSS DU731018-DU796676, DU800850-DU800864	DeLong et al., Science 2006 [18]	3,664.53
Hot500Mic10021006	microbial	Aquatic	Ocean	-	GSS DU731018-	DeLong et al., Science 2006 [18]	3,981.13

					DU796676, DU800850-DU800864		
Hot70Mic20021007	microbial	Aquatic	Ocean	-	GSS DU731018-DU796676, DU800850-DU800864	DeLong et al., Science 2006 [18]	2,157.18
Hot770Mic20031221	microbial	Aquatic	Ocean	-	GSS DU731018-DU796676, DU800850-DU800864	DeLong et al., Science 2006 [18]	2,597.30
HumanFecesSDVir	viral	Host-associated	-	-	GSS CC820769-CC821300	Breitbart et al., J. Bacteriol. 2003 [19]	-
KingLIMic20050821	microbial	Aquatic	Ocean	Kingman island	GP 28343	Dinsdale et al., PLoS One 2008 [12]	2,911.81
KingLIVir20050821	viral	Aquatic	Ocean	Kingman island	GP 28345	Dinsdale et al., PLoS One 2008 [12]	154.43
LeanMouseCecumMic2005	microbial	Host-associated	-	-	GP 17401	Turnbaugh et al., Nature 2006 [20]	3,144.82
LowSalternSDbayMic200407	microbial	Aquatic	Hypersaline	Low saltern 200407	GP 28359	Dinsdale et al., Nature 2008 [13]	4,564.80
LowSalternSDbayMic20051128	microbial	Aquatic	Hypersaline	Low saltern 200511	GP 28461	Dinsdale et al., Nature 2008 [13]	1,664.29
LowSalternSDbayVir200407	viral	Aquatic	Hypersaline	Low saltern 200407	GP 28353	Dinsdale et al., Nature 2008 [13]	67.90
LowSalternSDbayVir20051110	viral	Aquatic	Hypersaline	-	GP 28373	Dinsdale et al., Nature 2008 [13]	92.07
LowSalternSDbayVir20051128	viral	Aquatic	Hypersaline	Low saltern 200511	GP 28455	Dinsdale et al., Nature 2008 [13]	64.41
MarineBacterioplanktonS35131Euk	eukaryal	Aquatic	Ocean	-	CA BACTERIOPLANKTON_SMPL_S_35131	Hewson et al., Limnol Oceanogr 2009 [21]	804.70
MarineBacterioplanktonS35139Euk	eukaryal	Aquatic	Ocean	-	CA BACTERIOPLANKTON_SMPL_S_35139	Hewson et al., Limnol Oceanogr 2009 [21]	741.86
MarineBacterioplanktonS35147Euk	eukaryal	Aquatic	Ocean	-	CA BACTERIOPLANKTON_SMPL_S_35147	Hewson et al., Limnol Oceanogr 2009 [21]	809.65

MarineBacterioplanktonS35155Euk	eukaryal	Aquatic	Ocean	-	CA BACTERIOPLANKTON_SMPL_S_35155	Hewson et al., Limnol Oceanogr 2009 [21]	971.31
MarineBacterioplanktonS35163Euk	eukaryal	Aquatic	Ocean	-	CA BACTERIOPLANKTON_SMPL_S_35163	Hewson et al., Limnol Oceanogr 2009 [21]	1,055.38
MarineBacterioplanktonS35171Euk	eukaryal	Aquatic	Ocean	-	CA BACTERIOPLANKTON_SMPL_S_35171	Hewson et al., Limnol Oceanogr 2009 [21]	779.40
MarineBacterioplanktonS35179Euk	eukaryal	Aquatic	Ocean	-	CA BACTERIOPLANKTON_SMPL_S_35179	Hewson et al., Limnol Oceanogr 2009 [21]	864.10
MediterraneanBathypelagicEuk	eukaryal	Aquatic	Ocean	-	GSS EI942868-EI951915	Martin-Cuadrado et al., PLoS ONE 2007 [22]	-
MedSalternSDbayMic20051110	microbial	Aquatic	Hypersaline	Med saltern 20051110	GP 28377	Dinsdale et al., Nature 2008 [13]	4014.17
MedSalternSDbayMic20051111	microbial	Aquatic	Hypersaline	Med saltern	GP 28379	Dinsdale et al., Nature 2008 [13]	3,451.03
MedSalternSDbayMic20051116	microbial	Aquatic	Hypersaline	Med saltern 20051116	GP 28459	Dinsdale et al., Nature 2008 [13]	3,627.08
MedSalternSDbayMic20051128	microbial	Aquatic	Hypersaline	Med saltern 20051128	GP 28449	Dinsdale et al., Nature 2008 [13]	3,613.68
MedSalternSDbayVir20051110	viral	Aquatic	Hypersaline	Med saltern 20051110	GP 28375	Dinsdale et al., Nature 2008 [13]	72.77
MedSalternSDbayVir20051116	viral	Aquatic	Hypersaline	Med saltern 20051116	GP 28465	Dinsdale et al., Nature 2008 [13]	61.41
MedSalternSDbayVir20051122	viral	Aquatic	Hypersaline	Med saltern	GP 28445	Dinsdale et al., Nature 2008 [13]	56.80
MedSalternSDbayVir20051128	viral	Aquatic	Hypersaline	Med saltern 20051128	GP 28463	Dinsdale et al., Nature 2008 [13]	-
Mosq1SDVir20060125	viral	Host-associated	-	-	GP 28413	Dinsdale et al., Nature 2008 [13]	4.39
Mosq2SDVir2060606	viral	Host-associated	-	-	GP 28469	Dinsdale et al., Nature 2008 [13]	4.46
Move858Vir	viral	Aquatic	Ocean	Chesapeake Bay	GP 13694	Rush et al., PLoS Biol. 2007 [15]	-
MushroomHotSpringMic	microbial	Aquatic	Hot spring	-	GP 20953	Bhaya et al., ISME J., 2007 [23]	2,966.33
Norm3LungVir	viral	Host-	-	-	GP 39545	Willner et al., PLoS	61.23

20080407		associated				ONE 2009 [24]	
Norm4LungVir 20080407	viral	Host-associated	-	-	GP 39545	Willner et al., PLoS ONE 2009 [24]	20.51
Norm5LungVir 20080407	viral	Host-associated	-	-	GP 39545	Willner et al., PLoS ONE 2009 [24]	57.05
Norm6LungVir 20080407	viral	Host-associated	-	-	GP 39545	Willner et al., PLoS ONE 2009 [24]	49.29
Norm7LungVir 20080407	viral	Host-associated	-	-	GP 39545	Willner et al., PLoS ONE 2009 [24]	58.24
NutPorCompHawMic200602	microbial	Manipulated / Perturbed	-	Nutrient Pcomp	GP 28437	Vega Thurber et al., Env. Mic. 2009 [9]	2,083.43
NutPorCompHawVir200602	viral	Manipulated / Perturbed	-	Nutrient Pcomp	GP 28425	Vega Thurber et al., Proc. Nat. Acad. Sci. USA 2008 [10]	26.50
OctopusHotSpringMic	microbial	Aquatic	Hot spring	Yellowstone Octopus	GP 20953	Bhaya et al., ISME J., 2007 [17]	3,006.73
OctopusHotSpringVir	viral	Aquatic	Hot spring	Yellowstone Octopus	GP 20821	Pride et al., BMC Genomics 2008 [6]	-
OxMinZoneVir 200806-200	viral	Aquatic	Ocean	-	GP 40793	-	-
OxMinZoneVir 200806-90	viral	Aquatic	Ocean	-	GP 40791	-	93.13
PacificBeachSandEuk	eukaryal	Terrestrial	-	-	GP 13729	Naviaux et al., Mar Ecol Prog Ser 2005 [25]	-
PalmLIMic20050818	microbial	Aquatic	Ocean	Palmyra island	GP 28363	Dinsdale et al., PLoS One 2008 [12]	2,140.61
PalmLIVir20050818	viral	Aquatic	Ocean	Palmyra island	GP 28365	Dinsdale et al., PLoS One 2008 [12]	163.41
PANoVectorsBocasMic20050921	microbial	Host-associated	-	-	GP 28371	Wegley et al., Env. Mic. 2007 [26]	3,236.71
PAStromBahamasMic20050722	microbial	Aquatic	Microbialites	Pozas	GP 28385	Dinsdale et al., Nature 2008 [13]	4,504.51
PAStromCCMexVir20050722	viral	Aquatic	Microbialites	Pozas	GP 28355	Desnues et al., Nature 2008 [17]	17.26
PeruvianCoastalMarginMic	microbial	Aquatic	Ocean	-	SRA 001015	Biddle et al., Proc. Nat. Acad. Sci. USA 2008 [27]	2,521.81
pHPorCompHawMic200602	microbial	Manipulated / Perturbed	-	pH Pcomp	GP 28435	Vega Thurber et al., Env. Mic. 2009 [9]	2,178.71
pHPorCompHa	viral	Manipulated	-	pH Pcomp	GP 28423	Vega Thurber et al.,	39.92

wVir200602		/ Perturbed				Proc. Nat. Acad. Sci. USA 2008 [10]	
PrarieSoilKonzaVir	viral	Terrestrial	-	-	GSS ER781257-ER785833	Fierer et al., Appl. Environ. Microb. 2007 [11]	-
PrePondKentSTMic20060504	microbial	Aquatic	Fresh water	Kent SeaTech Pre 200605	GP 28407	Dinsdale et al., Nature 2008 [13]	4,049.11
PrePondKentSTVir20060504	viral	Aquatic	Fresh water	Kent SeaTech Pre 200605	GP 28411	Dinsdale et al., Nature 2008 [13]	47.00
RainforestSoilPeruVir	viral	Terrestrial	-	-	GSS ER781257-ER785833	Fierer et al., Appl. Environ. Microb. 2007 [11]	-
RicePaddySoilVir	viral	Terrestrial	-	-	GP 20811	Kim et al., Appl. Environ. Microb. 2008 [28]	-
RMStromCCMexMic20050722	microbial	Aquatic	Microbialites	Mexico	GP 28351	Dinsdale et al., Nature 2008 [13]	5,061.41
RMStromCCMexVir20050722	viral	Aquatic	Microbialites	Mexico	GP 28357	Desnues et al., Nature 2008 [13]	50.27
Rumen640FMic20051215	microbial	Host-associated	-	-	GP 28607	Dinsdale et al., Nature 2008 [13]	3,331.03
Rumen710FMic20051215	microbial	Host-associated	-	-	GP 28609	Dinsdale et al., Nature 2008 [13]	2,938.60
Rumen80FMic20051215	microbial	Host-associated	-	-	GP 28605	Dinsdale et al., Nature 2008 [13]	1,867.34
SaltonSea1Vir20060823	viral	Sediments	-	Salton Sea	GP 28613	Dinsdale et al., Nature 2008 [13]	88.67
SaltonSea2Vir20060823	viral	Sediments	-	Salton Sea	GP 28613	Dinsdale et al., Nature 2008 [13]	82.61
SaltonSeaMic20060823	microbial	Sediments	-	Salton Sea	GP 28613	Dinsdale et al., Nature 2008 [13]	3,806.14
SARVir20050630	viral	Aquatic	Ocean	Sargasso Sea	GP 17771	Angly et al., PLoS Biology 2006 [4]	19.95
SeawaterMissionBaySDVir	viral	Aquatic	Ocean	-	GSS BH898061-BH898933	Breitbart et al., Proc. Nat. Acad. Sci. USA 2002 [29]	-
SeawaterScrippsSDVir	viral	Aquatic	Ocean	-	GSS AY079522-AY080585	Breitbart et al., Proc. Nat. Acad. Sci. USA 2002 [29]	-
SedimentsMissionBaySDVir	viral	Sediments	-	-	GB CC821301-CC822456	Breitbart et al., Proc. R. Soc. B. 2004 [30]	-
SkanBayAlaska	viral	Aquatic	Ocean	-	GP 28619	Dinsdale et al.,	-

Vir20060927						Nature 2008 [13]	
SoilCC1Mic	microbial	Terrestrial	-	-	MG 4441690.3	-	6,063.12
SoilCP1Mic	microbial	Terrestrial	-	-	MG 4441689.3	-	5,927.76
SoilCP3Mic	microbial	Terrestrial	-	-	MG 4441691.3	-	5,776.93
SoilHF1Mic	microbial	Terrestrial	-	-	MG 4441642.3	-	5,887.77
SoilKP1Mic	microbial	Terrestrial	-	-	MG 4441994.3	-	5,952.04
SoilKP3Mic	microbial	Terrestrial	-	-	MG 4441643.3	-	5,777.56
SoilKW1Mic	microbial	Terrestrial	-	-	MG 4441664.3	-	5,741.19
SoilKW2Mic	microbial	Terrestrial	-	-	MG 4441691.4	-	5,766.49
SoilLF1Mic	microbial	Terrestrial	-	-	MG 4442455.3	-	6,181.55
SoilLF2Mic	microbial	Terrestrial	-	-	MG 4441644.3	-	6,131.82
SoilSJ1Mic	microbial	Terrestrial	-	-	MG 4441557.3	-	5,995.31
SoilSJ2Mic	microbial	Terrestrial	-	-	MG 4441645.3	-	5,974.06
SoilTF1Mic	microbial	Terrestrial	-	-	MG 4441688.3	-	6,119.44
SoilTF2Mic	microbial	Terrestrial	-	-	MG 4442452.3	-	6,312.57
SoilWF1Mic	microbial	Terrestrial	-	-	MG 4441556.3	-	5,812.88
SoilWF2Mic	microbial	Terrestrial	-	-	MG 4441665.3	-	5,632.30
SoilYN1Mic	microbial	Terrestrial	-	-	MG 4442453.3	-	5,960.67
SoilYN2Mic	microbial	Terrestrial	-	-	MG 4441687.3	-	5,899.56
SouthChinaSea SedimentsMic	microbial	Sediments	-	-	GP 33581	-	4,941.46
T0PortComHa wMic20060223	microbial	Host-associated	-	Coral tissue P. compressa	GP 28427	Vega Thurber et al., Env. Mic. 2009 [9]	3,618.00
T0PortComHa wVir20060223	viral	Host-associated	-	Coral tissue P. compressa	GP 28415	Vega Thurber et al., Proc. Nat. Acad. Sci. USA 2008 [10]	43.07
TempPorComp HawMic200602	microbial	Manipulated / Perturbed	-	Temperatur e Pcomp	GP 28431	Vega Thurber et al., Env. Mic. 2009 [9]	4,560.68
TempPorComp HawVir200602	viral	Manipulated / Perturbed	-	Temperatur e Pcomp	GP 28419	Vega Thurber et al., Proc. Nat. Acad. Sci. USA 2008 [10]	30.80
TilPondKentST Mic200511	microbial	Aquatic	Fresh water	Kent SeaTech Tpond 200511	GP 28387	Dinsdale et al., Nature 2008 [13]	4,204.22
TilPondKentST Mic20060504	microbial	Aquatic	Fresh water	Kent SeaTech Tpond 200605	GP 28405	Dinsdale et al., Nature 2008 [13]	4,007.80
TilPondKentST	microbial	Aquatic	Fresh water	Kent	GP 28603	Dinsdale et al.,	4,689.22

Mic200608				SeaTech Tpond 200608		Nature 2008 [13]	
TilPondKentSTVir20060504	viral	Aquatic	Fresh water	Kent SeaTech Tpond 200605	GP 28409	Dinsdale et al., Nature 2008 [13]	34.45
TilPondKentSTVir200608	viral	Aquatic	Fresh water	Kent SeaTech Tpond 200608	GP 28601	Dinsdale et al., Nature 2008 [13]	51.03
TpondKentSTVir200511	viral	Aquatic	Fresh water	Kent SeaTech Tpond 200511	GP 28361	Dinsdale et al., Nature 2008 [13]	35.71
WasecaFarmSoilMic	microbial	Terrestrial	-	-	GP 13699	Tringe SG et al., Science, 2005 [31]	5,350.40
XmasLIMic20050805	microbial	Aquatic	Ocean	Christmas island	GP 28347	Dinsdale et al., PLoS One 2008 [12]	3,483.52
XmasLIVir20050805	viral	Aquatic	Ocean	Christmas island	GP 28349	Dinsdale et al., PLoS One 2008 [12]	158.48

References:

1. Allen HK, Moe LA, Rodbumrer J, Gaarder A, Handelsman J (2008) Functional metagenomics reveals diverse beta-lactamases in a remote Alaskan soil. ISME J 3: 243-251.

2. Legault B, Lopez-Lopez A, Alba-Casado J, Doolittle WF, Bolhuis H, et al. (2006) Environmental genomics of "Haloquadratum walsbyi" in a saltern crystallizer indicates a large pool of accessory genes in an otherwise coherent species. BMC Genomics 7: 171.

3. Grzymski JJ, Murray AE, Campbell BJ, Kaplarevic M, Gao GR, et al. (2008) Metagenome analysis of an extreme microbial symbiosis reveals eurythermal adaptation and metabolic flexibility. Proceedings of the National Academy of Sciences 105: 17516-17521.

4. Angly FE, Felts B, Breitbart M, Salamon P, Edwards RA, et al. (2006) The marine viromes of four oceanic regions. PLoS Biology 4: e368.

5. Breitbart M, Haynes M, Kelley S, Angly F, Edwards RA, et al. (2008) Viral diversity and dynamics in an infant gut. Res Microbiol 159: 367-373.

6. Pride D, Schoenfeld T (2008) Genome signature analysis of thermal virus metagenomes reveals Archaea and thermophilic signatures. BMC Genomics 9: 420.

7. Bench SR, Hanson TE, Williamson KE, Ghosh D, Radosovich M, et al. (2007) Metagenomic characterization of Chesapeake Bay virioplankton. Appl Environ Microbiol 73: 7629-7641.

8. Qu A, Brulc JM, Wilson MK, Law BF, Theoret JR, et al. (2008) Comparative metagenomics reveals host specific metavirulomes and horizontal gene transfer elements in the chicken cecum microbiome. PLoS ONE 3: e2945.

9. Thurber RV, Willner-Hall D, Rodriguez-Mueller B, Desnues C, Edwards RA, et al. (2009) Metagenomic analysis of stressed coral holobionts. Environ Microbiol 11: 2148-2163.

10. Vega Thurber RL, Barott KL, Hall D, Liu H, Rodriguez-Mueller B, et al. (2008) Metagenomic analysis indicates that stressors induce production of herpes-like viruses in the coral Porites compressa. Proc Nat Acad Sci USA 105: 18413-18418.

11. Fierer N, Breitbart M, Nulton J, Salamon P, Lozupone C, et al. (2007) Metagenomic and small-subunit rRNA analyses reveal the genetic diversity of Bacteria, Archaea, Fungi, and viruses in soil. Appl Environ Microbiol 73: 7059-7066.

12. Dinsdale EA, Pantos O, Smriga S, Edwards RA, Angly F, et al. (2008) Microbial ecology of four coral atolls in the northern Line Islands. PLoS ONE 3: e1584.

13. Dinsdale EA, Edwards RA, Hall D, Angly F, Breitbart M, et al. (2008) Functional metagenomic profiling of nine biomes. Nature 452: 629-632.

14. Venter JC, Remington K, Heidelberg JF, Halpern AL, Rusch D, et al. (2004) Environmental genome shotgun sequencing of the Sargasso Sea. Science 304: 66-74.

15. Rusch DB, Halpern AL, Sutton G, Heidelberg KB, Williamson S, et al. (2007) The Sorcerer II global ocean sampling expedition: northwest Atlantic through eastern tropical Pacific. PLoS Biol 5: e77.

16. Woyke T, Teeling H, Ivanova NN, Huntemann M, Richter M, et al. (2006) Symbiosis insights through metagenomic analysis of a microbial consortium. Nature 443: 950-955.

17. Desnues C, Rodriguez-Brito B, Rayhawk S, Kelley S, Tran T, et al. (2008) Biodiversity and biogeography of phages in modern stromatolites and thrombolites. Nature 452: 340-343.

18. DeLong EF, Preston CM, Mincer T, Rich V, Hallam SJ, et al. (2006) Community genomics among stratified microbial assemblages in the ocean's interior. Science 311: 496-503.

19. Breitbart M, Hewson I, Felts B, Mahaffy JM, Nulton J, et al. (2003) Metagenomic analyses of an uncultured viral community from human feces. J Bacteriol 185: 6220-6223.

20. Turnbaugh PJ, Ley RE, Mahowald MA, Magrini V, Mardis ER, et al. (2006) An obesity-associated gut microbiome with increased capacity for energy harvest. Nature 444: 1027-131.

21. Hewson I, Paerl RW, Tripp HJ, Zehr JP, Karl DM (2009) Metagenomic potential of microbial assemblages in the surface waters of the central Pacific Ocean tracks variability in oceanic habitat. Limnol Oceanogr 54: 1981-1994.

22. Martín-Cuadrado A, López-García P, Alba J, Moreira D, Monticelli L, et al. (2007) Metagenomics of the deep mediterranean, a warm bathypelagic habitat. PLoS ONE 2: e914.

23. Bhaya D, Grossman AR, Steunou A, Khuri N, Cohan FM, et al. (2007) Population level functional diversity in a microbial community revealed by comparative genomic and metagenomic analyses. ISME J 1: 703-713.

24. Willner D, Furlan M, Haynes M, Schmieder R, Angly FE, et al. (2009) Metagenomic analysis of respiratory tract DNA viral communities in cystic fibrosis and non-cystic fibrosis Individuals. PLoS ONE 4: e7370.

25. Naviaux RK, Good B, McPherson JD, Steffen DL, Markusic D, et al. (2005) Sand DNA - a genetic library of life at the water's edge. Mar Ecol Prog Ser 301: 9-22.

26. Wegley L, Edwards R, Rodriguez-Brito B, Liu H, Rohwer F (2007) Metagenomic analysis of the microbial community associated with the coral Porites astreoides. Environmental Microbiology 9: 2707-2719.

27. Biddle JF, Fitz-Gibbon S, Schuster SC, Brenchley JE, House CH (2008) Metagenomic signatures of the Peru Margin subseafloor biosphere show a genetically distinct environment. Proc Nat Acad Sci USA 105: 10583-10588.

28. Kim K, Chang H, Nam Y, Roh SW, Kim M, et al. (2008) Amplification of uncultured single-stranded DNA viruses from rice paddy soil. Appl Environ Microbiol 74: 5975-5985.

29. Breitbart M, Salamon P, Andresen B, Mahaffy J, Segall A, et al. (2002) Genomic analysis of uncultured marine viral communities. Proc Nat Acad Sci USA 99: 14250-14255.

30. Breitbart M, Felts B, Kelley S, Mahaffy JM, Nulton J, et al. (2004) Diversity and population structure of a near-shore marine-sediment viral community. Proc R Soc B 271: 565–574.

31. Tringe SG, von Mering C, Kobayashi A, Salamov AA, Chen K, et al. (2005) Comparative metagenomics of microbial communities. Science 308: 554-557.
