# Supplementary figures and images for: The GAAS Metagenomic Tool and Its Estimations of Viral and Microbial Average Genome Size in Four Major Biomes
Source: PLoS Comput Biol. 2009 Dec 11;5(12):e1000593. doi: 10.1371/journal.pcbi.1000593 (PMC2781106; doi:10.1371/journal.pcbi.1000593)

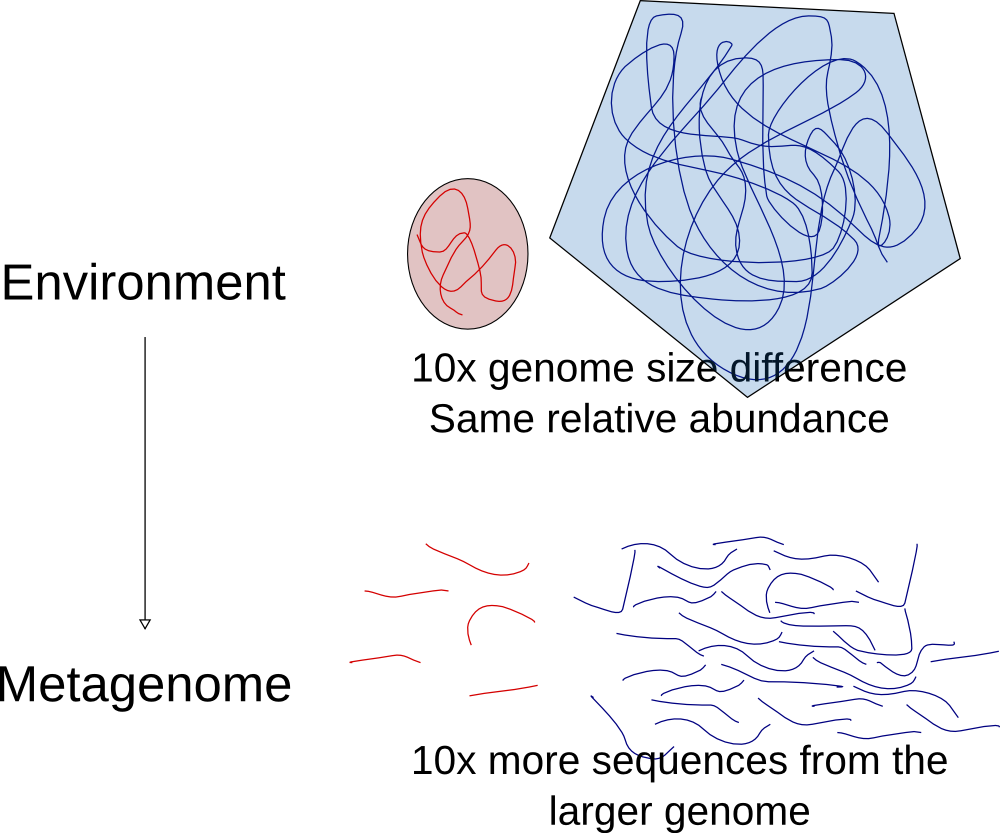

Supplement: Figure S1 — Sampling bias toward larger genomes in metagenomic libraries. Larger genomes will produce more fragments of a given size, and are more likely to be sampled even if they occur in the same abundance as small genomes. (0.17 MB TIF) [file pcbi.1000593.s004.tif]

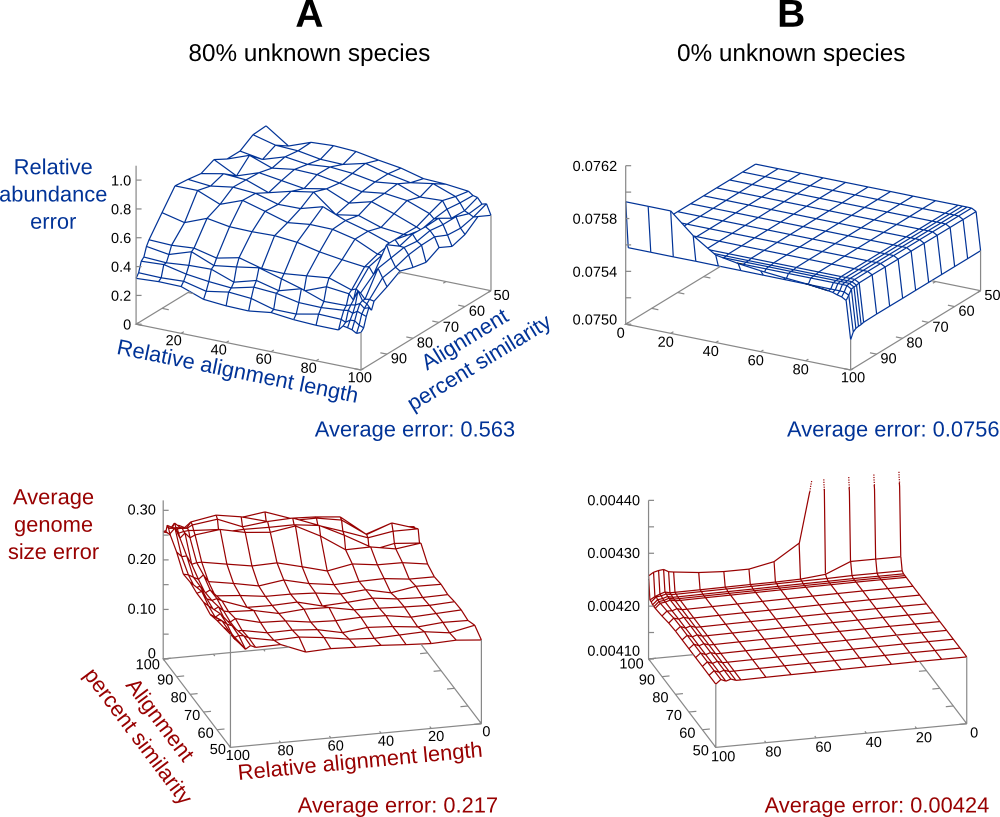

Supplement: Figure S2 — Accuracy of the GAAS estimates when no species are unknown. Error on the relative abundance (top) and average genome size estimates (bottom) when: (A) 80% of the species were treated as unknown, (B) no species were assumed to be unknown. The simulated viromes were made of 100 bp sequences. (0.29 MB TIF) [file pcbi.1000593.s005.tif]

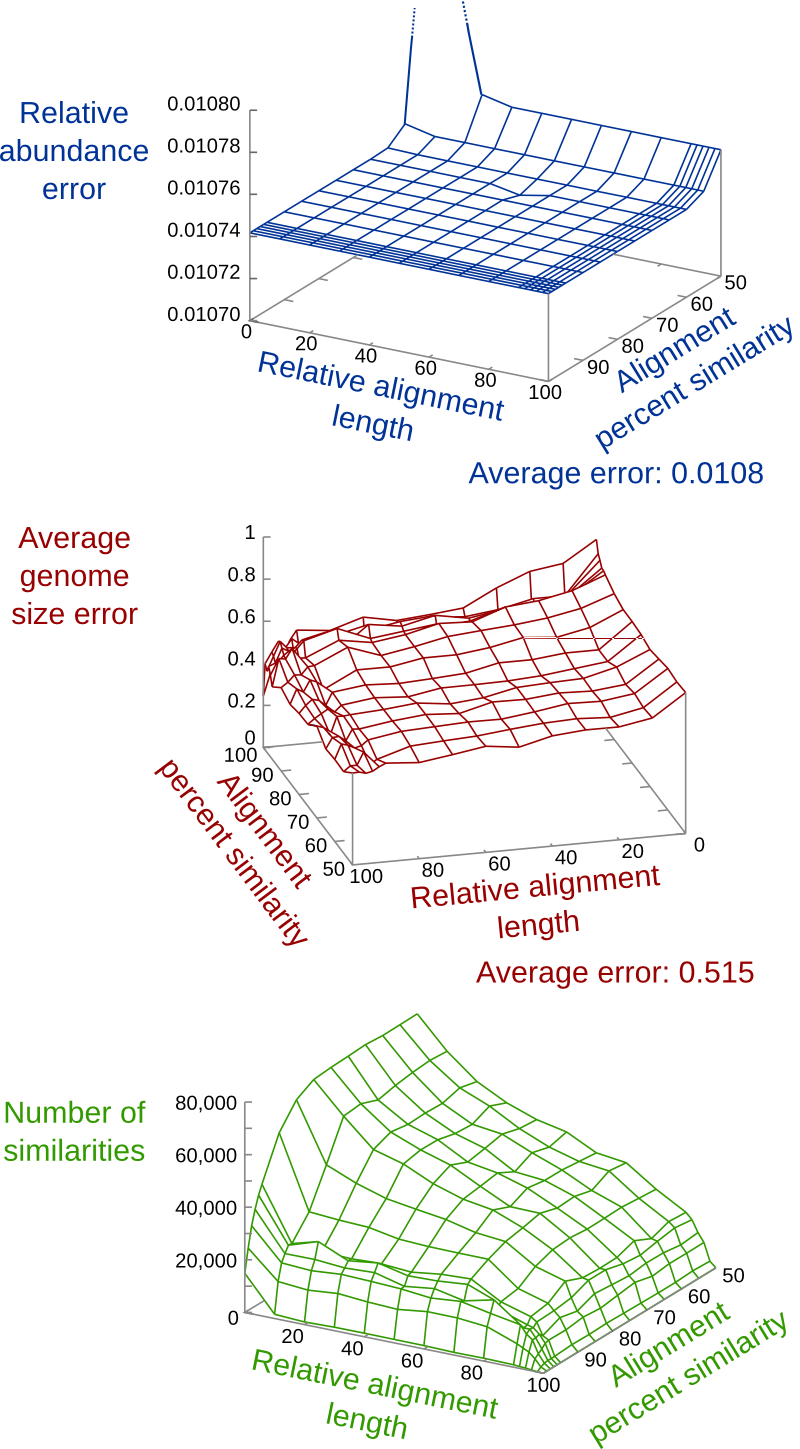

Supplement: Figure S3 — Accuracy of GAAS estimates for microbial metagenomes. GAAS relative abundance error (top), average genome size error (middle) and number of similarities (bottom) for the JGI simulated microbial metagenomes (∼1,200 bp/read). 80% of the species were treated as unknown. (0.39 MB TIF) [file pcbi.1000593.s006.tif]

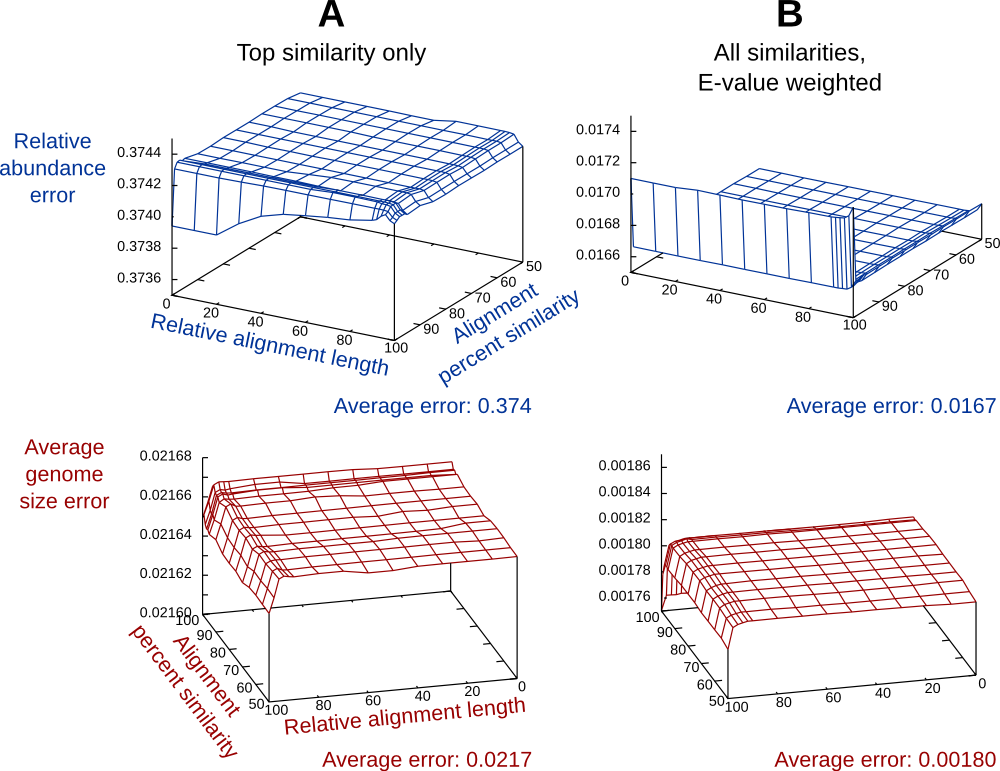

Supplement: Figure S4 — Effect of using all similarities for microbial strains. The error on community composition (top) and average genome length (bottom) for simulated metagenomes made of 15 Escherichia coli strains was estimated by GAAS. Sequence length was 100 bp and no strains were treated as unknown. (0.27 MB TIF) [file pcbi.1000593.s007.tif]

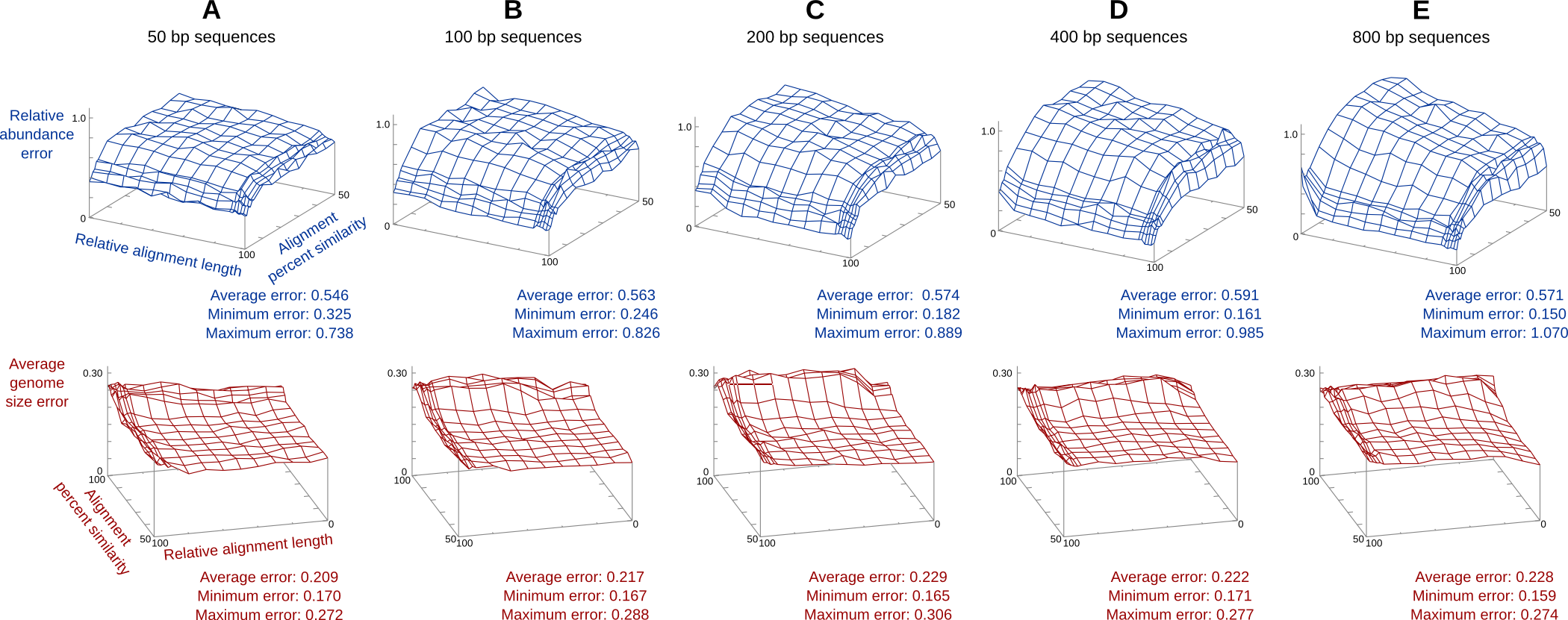

Supplement: Figure S5 — Effect of metagenomic sequence length on the accuracy of GAAS estimates. Error was calculated for the relative abundance (top) and average genome length (bottom) estimates. 80% of the species in the viral simulated metagenomes were treated as unknown. (0.64 MB TIF) [file pcbi.1000593.s008.tif]

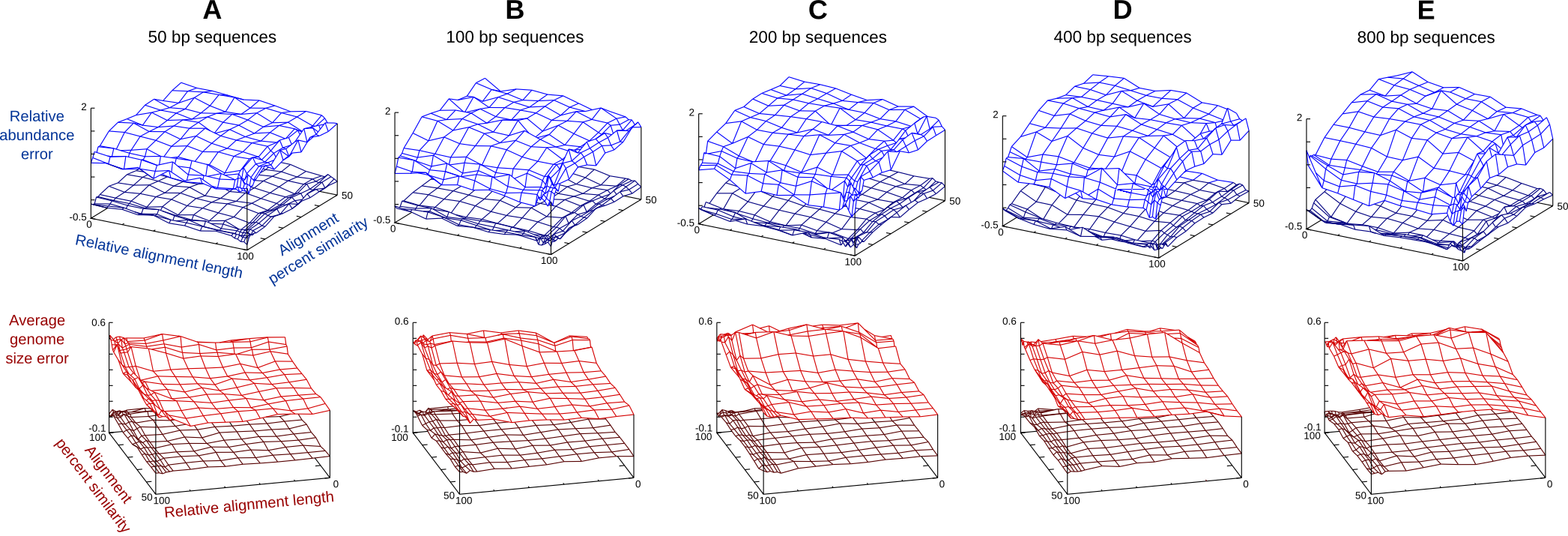

Supplement: Figure S6 — Error surfaces for Figure S5. The two surfaces of each graph correspond to the average error ± the standard deviation for the >1,200 simulated metagenomes. (0.62 MB TIF) [file pcbi.1000593.s009.tif]

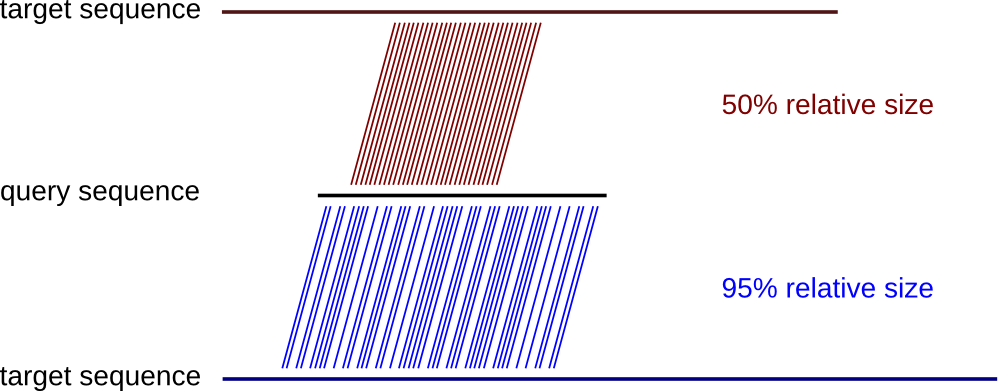

Supplement: Figure S7 — The relative alignment length filtering parameter. The relative alignment length is defined as the ratio of the length of the alignment over the length of the query sequence length, expressed in percent. (0.14 MB TIF) [file pcbi.1000593.s010.tif]
